# Is stroke incidence increased in survivors of adult cancers? A systematic review and meta-analysis

**DOI:** 10.1007/s11764-021-01122-7

**Published:** 2021-11-05

**Authors:** Melanie Turner, Peter Murchie, Sarah Derby, Ariel Yuhan Ong, Lauren Walji, David McLernon, Mary-Joan Macleod, Rosalind Adam

**Affiliations:** 1 Institute of Applied Health Sciences, University of Aberdeen, Polwarth Building, Foresterhill, Aberdeen, AB25 2ZD UK; 2 Institute of Cancer Sciences, University of Glasgow, Wolfson Wohl Cancer Research Centre, Bearsden, Glasgow, G61 1BD UK; 3 Oxford Eye Hospital, Level Lg1 John Radcliffe Hospital, Headley Way, Headington, Oxford, OX3 9DU UK; 4 University of Aberdeen Medical School, Polwarth Building, Foresterhill, Aberdeen, AB25 2ZD UK; 5 Institute of Medical Sciences, University of Aberdeen, Foresterhill, Aberdeen, AB25 2ZD UK

**Keywords:** Stroke, Survivorship, Adult cancer, Systematic review

## Abstract

**Purpose:**

Existing research hints that people living with and beyond cancer are at an increased risk of stroke. However, there is insufficient evidence to appropriately inform guidelines for specific stroke prevention or management for cancer patients. We conducted a systematic review and meta-analysis to describe and quantify stroke incidence in people living with and beyond cancer.

**Methods:**

Medline, CINAHL, and EMBASE were searched for epidemiological studies comparing stroke incidence between cancer and non-cancer patients. Reviewers independently extracted data; random-effects meta-analyses and quality assessment were performed.

**Results:**

Thirty-six studies were narratively synthesised. Meta-analysis was conducted using seven studies. Methodological quality was high for most studies. Study populations were heterogeneous, and the length of follow-up and risk factors varied. There was a variation in risk between different cancer types and according to stroke type: pancreatic (HR 2.85 (95% CI 2.43–3.36), ischaemic) (HR 2.28 (95% CI 1.43–3.63), haemorrhagic); lung (HR 2.33 (95% CI 1.63–3.35), ischaemic) (HR 2.14 (95% CI 1.45–3.15), haemorrhagic); and head and neck (HR 1.54 (95% CI 1.40–1.69), haemorrhagic) cancers were associated with significantly increased incidence of stroke. Risk is highest within the first 6 months of diagnosis. Narrative synthesis indicated that several studies also showed significantly increased incidence of stroke in individuals with colorectal cancer, breast cancer, ovarian cancer, nasopharyngeal cancer, leukaemia, and myeloma, and those who have received radiotherapy for head and neck cancers and platinum-based chemotherapy may also have higher stroke incidence.

**Conclusions:**

Stroke incidence is significantly increased after diagnosis of certain cancers.

**Implications for Cancer Survivors:**

Cardiovascular risk should be assessed during cancer survivorship care, with attention to modifying shared cancer/cardiovascular risk factors.

**Supplementary Information:**

The online version contains supplementary material available at 10.1007/s11764-021-01122-7.

## Background

Advances in cancer treatments have improved cancer survival in recent decades with approximately half of patients diagnosed with cancer in developed countries now expected to survive for at least 10 years [1]. The population of cancer survivors in the UK is projected to increase by approximately one million per decade from 2010 to 2040. Cancer disproportionately affects older people, almost a quarter of people aged 65 and over are cancer survivors [2]. The relationship between cancer and other major conditions thus becomes increasingly important.

Many studies have suggested that people with a history of some, but not all, cancers are at increased risk of cardiovascular disease [3]. Cancer is associated with a substantially increased 6-month risk of arterial thromboembolism, including ischaemic stroke [4–7]. Furthermore, cancer may increase the risk of early deterioration, disability, recurrent thromboembolism, and mortality after stroke [8–10]. Stroke risk has been shown to vary by age, gender, cancer type, histology, stage, and time after diagnosis [11] and to remain elevated for up to 10 years following cancer diagnosis [4, 12].

Any actual increased risk of stroke associated with cancer is likely to be multifactorial. Individuals with cancer may suffer strokes caused by mechanisms related to malignancy-associated prothrombotic states including changes in platelet function and increased production of neutrophil elastase traps (NETs); cardiotoxicity-associated with chemotherapeutic and radiotherapy treatments; shared risk factors; detection bias due to intensified surveillance; and underutilisation and frequent interruption of antithrombotic agents because of bleeding concerns and the potential for haemorrhagic stroke [4, 13].

Because evidence is lacking, there are currently no specific guidelines for stroke prevention, identification, or management in patients living with and beyond cancer. Currently, there is no tailored approach to preventing, managing, and treating patients with both cancer and stroke, a fact which could be worsening long-term outcomes for people with cancer. The lack of high-quality evidence to support optimal practice is likely to contribute to significant variations in clinical practice in secondary care. Optimal clinical practice in the prevention, diagnosis, and treatment of stroke in those living with and beyond cancer is crucial to improve survival and long-term outcomes.

There is a need to consolidate the evidence on the incidence of stroke in individuals living with and beyond cancer due to the potentially synergistic impact on stroke severity and disease outcomes. Our objective was to conduct a systematic review and meta-analysis of epidemiological studies comparing stroke incidence between individuals living with and beyond cancer and those without cancer (matched controls, controls from the general population, or using the general reference population of a region or country).

## Methods

A systematic review was conducted to identify observational studies and randomised controlled trials (RCTs) of stroke incidence in patients living with and beyond cancer. The review was conducted according to the Cochrane Handbook and the Preferred Reporting Items for Systematic Review and Meta-Analyses (PRISMA) statement [14]. A review protocol was registered and is available at https://www.crd.york.ac.uk/prospero/display_record.php?RecordID=58953.

### Search strategy

Keywords and Boolean operators were explored and combined on the advice of a senior medical librarian to search the electronic databases MEDLINE, CINAHL, and EMBASE. They were searched for quantitative studies published between 1946 and 2021 which explored the relationship between cancer and stroke risk. Database searches took place in 2017, 2018, 2020, and 2021. The last date of a search was 3rd February 2021. An example of the detailed search strategy is shown in Supplementary Fig. 1. Reference lists of reviews of stroke and cancer and all relevant full-text papers included in this review were searched for additional relevant titles.

### Inclusion and exclusion criteria

This review considered all types of observational studies and RCTs in which ischaemic stroke, haemorrhagic stroke, or transient ischaemic stack (TIA) incidence was compared between individuals living with and beyond cancer and a control group of people never diagnosed with any cancer. Studies were eligible for inclusion if they were population-based cohort studies of adults who had received a diagnosis of cancer and had the onset of stroke or TIA as an observation endpoint. Excluded studies were other systematic reviews, case studies with less than 10 people, qualitative studies, letters, or editorials, not published in English, or focused on non-melanoma skin cancer. Excluded studies were documented with reasons for their exclusion. We initially included adult survivors of childhood cancers in the inclusion criteria but subsequently excluded these due to them being a different entity from adult-onset cancers and their heterogeneity.

### Study selection

Study titles and abstracts were screened independently by three authors (MT, SD, AYO). Full texts were retrieved for all relevant abstracts and independently reviewed against the inclusion and exclusion criteria by at least two authors (MT, SD, AYO). Disagreements regarding study eligibility were resolved by discussion.

### Data extraction

A data extraction form was created in Microsoft Word, and an Excel file was used to collate the extracted data. Data extracted included year of publication, cancer site, control group, age profile, primary and secondary outcomes, adjustment for covariates, handling of prior stroke, study size, and follow-up. The primary endpoint was occurrence of stroke, TIA, or cerebrovascular accident as defined by each individual study. Extraction was carried out by two reviewers (MT, RA). MT had read all the studies and compiled and reviewed all extraction forms for consistency.

### Study quality assessment

Quality assessment was assessed independently by three authors (MT, RA, LW). The Newcastle–Ottawa Scale (NOS) was utilised for the quality assessment of the included studies [15]. NOS scale rates observational studies based on 3 parameters: selection of study population, comparability between the exposed and unexposed groups, and exposure/outcome assessment and follow-up. It assigns a maximum of 4 stars for selection and representativeness of patients, 2 stars for comparability between patient groups, and 3 stars for exposure/outcome assessment and follow-up. Studies with less than a total of 5 stars were considered low quality, while 5–6 stars reflected moderate quality, and more than 7 stars indicated high quality. Inter-rater reliability was assessed using Cohen’s kappa statistic, calculated on SPSS version 25 software.

Heterogeneity was assessed using visual inspection of forest plots, Cochran Q tests, and the *I*^2^ statistic as a measure for inconsistency due to chance.

### Data synthesis and meta-analysis

We adopted a narrative approach to describing the number of studies, study settings, proportion of sex, mean or median age, and covariates adjusted for in each study. Our primary outcome was incidence of stroke in patients living with and beyond cancer.

For our quantitative assessment of stroke incidence in patients living with and beyond cancer, we selected the final adjusted statistical model from each article. Fully adjusted models considered a variety of confounding factors. Outcome data (hazard ratios) from included studies were entered into Review Manager version 5.3. Studies which reported hazard ratios (HR) were combined in a meta-analysis. If HRs were not provided, they were calculated manually from relevant information, e.g. estimate(beta), standard error, or confidence intervals.

Meta-analyses were performed on the log hazards of stroke incidence to provide the overall hazard ratio (HR) and confidence interval (CI) for individual cancer types (lung, pancreatic, stomach, ovarian, and head and neck) for ischaemic stroke or haemorrhagic stroke only depending on the number of studies and the data available. We chose to perform a random-effects (using the DerSimonian and Laird approach) meta-analysis of hazard ratios which account for both patient events and the time to events, and this effect estimate was measured in the largest proportion of included studies (*n* = 25, 70%). The control group was taken as the reference category. Statistical heterogeneity was assessed using the *I*^2^ statistics.

## Results

### Database searches

**Fig. 1:**
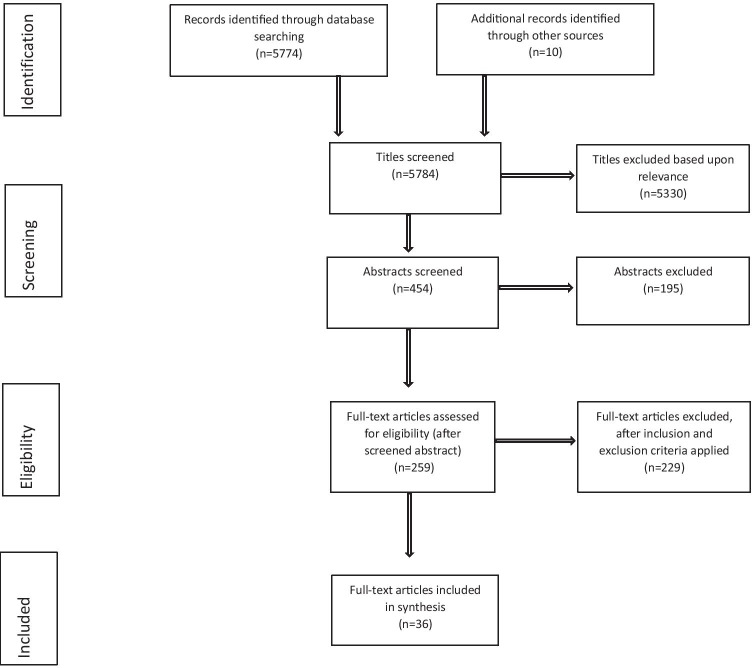
Flowchart of study selection

A flow diagram of the study selection process is presented in Fig. 1. We carried out searches in 2017, 2018, and 2020 and updated the search on 3rd February 2021. A total of 5784 articles were identified from database searches. A total of 454 abstracts were screened, and 259 full-text articles were assessed, of which 36 satisfied the eligibility criteria and were included in the systematic review.

### Definitions of stroke

Multiple definitions of stroke were used (Supplementary Table 1). The majority of these (*n* = 26) were based on ICD-9-CM and ICD-10 codes. Two studies were not clear on their definition of stroke (28, 33).

### Characteristics of included studies

An overview of the characteristics of the studies included in the analysis is presented in Table 1.

**Table 1:** Characteristics of included studies

Study (author, year, reference)	Data source; location; time period	Cancer site(s)	Control group	Age profile	Specific outcomes investigated (primary and secondary)	Adjustment for covariates	Handling of prior stroke	Lag from cancer diagnosis to follow-up	Study size	Average follow-up	Range of follow-up duration
Adelborg et al., 2019 [16]	Danish Cancer Registry, Denmark; Jan 2000–Nov 2013	Haematological cancers	General population controls matched in a 5:1 ratio on age, sex, previous thromboembolic events, bleeding, and solid cancer	Range 15–70+ yr	Primary outcome = myocardial infarction, stroke, venous thromboembolism , and bleeding requiring hospital contact	Not stated	Adjusted for in matching	None	32,242 cases; 160,252 controls	Median 2.5 yr	0–10 yr
Armenian et al., 2016 [17]	Kaiser Permanente Southern California members; USA; 2000–2007	Any cancer 14 specific sites	Age, sex, and region-matched cancer-free controls	Median 60 yr (range 40–96)	Primary outcome = incidence rates for CVD (composite); plus exploratory analysis of cardiomyopathy/heart failure, ischaemic heart disease, stroke Secondary outcomes = survival analysis, all-cause mortality	Age, sex, race/ethnicity, smoking, overweight/obesity; time-updated diabetes, hypertension, dyslipidaemia	Those with any prior CVD excluded	2 yr	36,232 exposed; 73,545 controls	4.4 y (exposed); 4.5y (controls)	0–8 yr
Andersen et al., 2018 [18]	Danish Cancer Registry; Denmark; Jan 2004–July 2012	15 most common cancers related and unrelated to smoking	Population controls matched 10:1 on sex, date of birth, income, and education	Cancer population: mean = 66.6 yr (SD 13.55) Control population: mean = 66.49yr (SD 13.43)	Primary outcome = hospitalisation for stroke	Age, sex, education, and disposable income	Analysed separately. Two groups: stroke diagnosed in year before cancer diagnosis and stroke diagnosed in the year following cancer diagnosis	None	258,721 exposed; 2,496,184 controls	Not stated	0–1 yr
Chang et al., 2013 [19]	Taiwan National Health Insurance database; Taiwan; 2000–2008	Cervical cancer	Four cancer-free controls to each cancer case; matched on cervical cancer-month, cervical cancer-year, and age (each 5 years)	20+ yr	Diagnosis of stroke (ICD-9-CM 430–438) at discharge and follow-up to end of 2009 Incidence and HRs of risk of stroke Primary outcome = incidence of stroke	Age, urbanisation level, diabetes, and hypertension	Those with history of stroke prior to index date excluded	≥ 3 yr	20,286 exposed; 81,144 controls	Not stated	0– ≥ 3 yr
Chan et al., 2018 [20]	Taiwan National Health Insurance Research Database; Taiwan; 2000–2009	Pancreatic cancer	Cancer-free controls randomly selected from population matched by propensity score with sample size fourfold of cancer cohort	Mean age 65 yr (SD 13.6)	Primary outcome = occurrence of stroke	Sex, age, hypertension, diabetes, hyperlipidaemia, coronary artery disease, and atrial fibrillation	Those with history of stroke prior to index date excluded	None	7479 exposed; 29,916 controls	Not stated	0–3 yr
Chen et al., 2011 [21]	Taiwan National Health Insurance database; Taiwan; 1999–2007	Lung cancer	Cancer-free controls matched 2:1 on age, sex, and month of lung cancer diagnosis	Mean age 67 yr (SD 12.3)	Primary outcome = occurrence of stroke	Age, level of urbanisation, history of hypertension, diabetes, coronary heart disease, atrial fibrillation and chronic obstructive pulmonary disease	Those with history of stroke prior to index date excluded (except for traumatic strokes)	None	52,089 exposed; 104,178 controls	Exposed group median 0.7 yr; control group median 4.1 yr	Not stated
Chia et al., 2013 [22]	National Cancer Institute’s (NCI) Surveillance, Epidemiology, and End Results (SEER)-Medicare database; USA; 1998–2002	Ovarian cancer	Cancer-free controls randomly matched based on country of residence	66–80 + yr	Primary outcome = incidence of comorbid conditions following cancer diagnosis	Age, race, or ethnicity	Those with history of comorbid condition not included in incidence rate estimate	None	5087 exposed; 5087 controls	Not stated	0–1 yr
Chu et al., 2011 [23]	Taiwan National Health Insurance database; Taiwan; 2000–2002	Head and neck cancer	Four controls to each cancer case; matched for gender, age, comorbidity (hypertension, diabetes mellitus, or both)	Median 50.1 yr	Primary outcome = incidence of stroke	Gender, age, hypertension, diabetes mellitus, and years after index date	Those with prior stroke excluded	None	13,390 exposed; 53,517 controls	Not stated	0– ≥ 8 yr
Chu et al., 2013 [24]	Taiwan National Health Insurance database; Taiwan; 2000–2003	Nasopharyngeal cancer	Insured general population without cancer or stroke history. Matched on sex, age and year	20 to > 60	Primary outcome = incidence of stroke	Sex, age, comorbidities (hypertension, diabetes mellitus, atrial fibrillation, hyperlipidaemia, alcoholism	Those with prior stroke excluded	None	4615 exposed; 36,919 controls	Median 6.63 yr	0–10 yr
De Bruin et al., 2009 [25]	Patients treated at 4 cancer centres or university hospitals; Netherlands; diagnosed between 1965 and 1995. Follow-up collected between 2004–2008	Hodgkin lymphoma	General population of Netherlands	Younger than 51 years at time of cancer diagnosis	Primary outcome = incidence of stroke. Secondary outcomes = risk factors for stroke	Cumulative incidence adjusted for competing risk of death	Those with prior stroke excluded	5 yr	2201 exposed; general population used as controls	Median 17.5 yr	5–38 yr
Dorresteijn et al., 2001 [26]	Patients treated at Netherlands Cancer Institute/Antoni van Leeuwenhoekhuis; Netherlands; 1977–1998	Head and neck cancer	General population of Netherlands	< 60yr. Median age at treatment 49.3 yr	Primary outcome = incidence of stroke. Secondary outcomes = risk factors for stroke	Age, sex	Not stated	0.5 yr	367 exposed; general population used as controls	Median 7.8 yr	0.5–22.6 yr
Haynes et al., 2002 [27]	PROCLIPS electronic database maintained by Department of Radiation Oncology of University of Pennsylvania, USA; 1987–2000	Head and neck cancer	Expected incidence based on population data from Stockholm, Sweden (Ref 17 in study)	< 80 yr	Primary outcome = rate of stroke	Age, gender, smoking status	Not stated	None	413 exposed; general population used as controls	Not stated	2–146 months after radiotherapy
Hooning et al., 2006 [28]	Late Effects Cancer cohort collected in the Netherlands Cancer Institute or the Erasmus MC, Daniel den Hoed Cancer Centre; Netherlands; 1970–1986. Follow-up until 2000	Breast cancer	Dutch female population	Median age 49 yr	Primary outcome = incidence of stroke. Secondary outcomes = treatment effects on stroke risk	Age	Not stated	10 yr	4368 exposed; general population used as controls	Median 17.7 yr	10– ≥ 20 yr
Kuan et al., 2014 [29]	Taiwan National Health Insurance database; Taiwan; 2003–2011	Ovarian cancer	Cancer-free controls matched 1:1 on age, gender, time or enrolment; comorbidities related to cerebrovascular events	Median age 49 yr (IQR 41–58)	Primary outcome = occurrence of ischaemic stroke	Age, sex, and comorbidities	Those with prior stroke excluded	None	8810 exposed; 8810 controls	Median 2.68 yr (IQR 1.44–4.98 yr) for exposed; median 3.85 yr (IQR 1.83–6.14 yr) for controls	0–9 yr
Kuan et al., 2015 [30]	Taiwan National Health Insurance database; Taiwan; 2003–2011	Gastric cancer	Cancer-free controls matched 1:1 on age, gender, time or enrolment; comorbidities related to ischaemic stroke	Median age 65 yr (IQR 53–76)	Primary outcome = incidence of ischaemic stroke. Secondary outcome = risk factors for ischaemic stroke	Age, sex, and comorbidities	Those with prior stroke excluded	None	22,530 exposed; 22,530 controls	Not stated	0–9 yr
Lauritsen et al., 2019 [31]	Danish Testicular Cancer Database; Denmark; 1984–2007	Unilateral testicular cancer or germ cell cancer (GCC) of extragonadal origin	Cancer-free controls matched 1:10 on no prior cancer and date of birth	Controls: median = 34.2 yr (IQR 27.9–41.9) Cases: surveillance group median = 34.2 yr (IQR 28.3–41.8) Radiotherapy group median = 38.6 yr (IQR 32.4–45.6) BEP group median = 35.4 yr (IQR 26.9–43.5) MTOL group median = 35.4 yr (26.9–43.5)	Primary outcomes = cardiovascular risk factors, incident cerebrovascular accident (CVA), and cardiovascular death	Age	Those with prior CVA excluded	None	5185 exposed; 51,848 controls	Not stated	0–5 yr
Lee et al., 2011 [32]	Taiwan National Health Insurance database; Taiwan; 1997–1998; follow-up to 2008	Nasopharyngeal cancer	Patients hospitalised for an appendectomy between 1997 and 1998, matched 4:1 with gender and age (35–44, 45–54, and 55–64 yr)	35–64 yr	Primary outcome = incidence of ischaemic stroke	Age, gender, hypertension, diabetes coronary artery disease, hyperlipidaemia, atrial fibrillation, socioeconomic status, region, and urbanisation of residence	Not stated	None	1094 exposed; 4376 controls	Not stated	0–12 yr
Maduro et al., 2010 [33]	Patients treated at University Medical Centre Groningen; Netherlands; 1989–2002; follow-up to 2007	Cervical cancer	Dutch female population	Median age 50.7 yr Range 20.9–85.9 yr	Primary outcome = incidence of cardiovascular events (MI, AP, CHF, CVA, peripheral arterial disease, venous thromboembolic events, and others. Secondary outcomes = risk factors for MI, AP or CHF	Age, sex, and calendar period-specific for expected incidence rates	Patients with pretreatment cardiovascular events included. Not clear if adjustment made when calculating SIRs	None	277 exposed; general population used as controls	Median 4.5 yr	0.1–17.0 yr
Melloni et al., 2017 [34]	ARISTOTLE trial; 1000 centres in 40 countries; 2006–2011	Any cancer, excluding basal or squamous cell skin cancer	Trial patients who had no cancer	Active cancer: median age 74 yr (IQR 68–80) Remote cancer: median age 75 yr (IQR 69–80) No cancer: 70 yr (IQR 62–76)	Primary outcome = ischemic outcomes (stroke or systemic embolism, ischaemic stroke, myocardial infarction, death from ischaemic cause). Bleeding outcomes (ISTH major bleeding, major or CRNM bleeding, any bleeding, intracranial bleeding)	Age, region, weight, diabetes, hypertension, moderate valvular disease, prior stroke/TIA/systemic embolism, type of atrial fibrillation, and prior vitamin K antagonist	Adjusted for in outcome	None stated	Active cancer = 157; remote cancer = 1079; Non-cancer controls = 16,947	Not stated in publication. From trial publication: maximum follow-up 4 yr; median 1.8 yr	Not stated in publication. 0–4 yr in trial
Moser et al., 2006 [35]	European Organisation of Research and Treatment of Cancer database; Netherlands or Belgium patients; 1980–1999. Person-time analysis for stroke started from 1985 to 2001	Non-Hodgkin lymphoma	General Dutch population derived from the Continuous Morbidity Registration of the Department of General Practice (GP) at the University of Nijmegen (CMRN)	15–85 yr	Primary outcome = incidence of cardiovascular disease (categories were chromic heart failure, coronary artery disease, myocardial infarction, angina pectoris, stroke). Secondary outcomes = treatment effects on risk of cardiovascular disease	Incidences categorised by age, sex and calendar period in both study and CMRN cohort	Patients with history of cardiovascular disease excluded, except patients with pre-existing hypertension	0.5 yr	476 exposed; general population used as controls	Median 8.4 yr	0.5–16 yr for stroke
Moutsten et al., 2019 [36]	Danish prospective Diet, Cancer and Health cohort; Denmark; 1993–2013	Prostate cancer	Those without cancer (cancer-free) in the diet, cancer, and health cohort	50–64 yr	Primary outcome = incident acute MI, IS, or HF	Year of study entry, education, smoking, alcohol intake, physical activity, BMI, and waist circumference, blood pressure, diabetes, and serum cholesterol. MI and HF included as competing events along with deaths from other causes	Excluded	None	1546 cases; 25,436 controls	Median 18.5 yr	Not clear
Navi et al., 2015 [4]	SEER-Medicare dataset; USA; 2001–2009	Breast, colorectal, lung, pancreatic, prostate cancer	Matched cancer-free controls from cohort of fee-for-service Medicare enrolees. Matched on year of birth, sex, race, SEER registry, and CCI	Mean age (SD) Breast cohort = 76 yr (7) Colorectal cohort = 78 yr (7) Lung cohort = 76 yr (7) Pancreatic cohort = 78 yr (7) Prostate cohort = 75 yr (6)	Primary outcome = composite of ischaemic or haemorrhagic stroke Secondary outcomes = ischaemic stroke alone and haemorrhagic stroke alone	None stated	Not stated	None	327,389 pairs of cases and controls	Median follow-up 4.3 yr versus 4.7 yr for breast cancer; 2.9 yr versus 4.5 yr for colorectal cancer; 0.6 yr versus 4.5 yr for lung cancer; 0.3 yr versus 4.3 yr for pancreatic cancer; 4.5 yr versus 4.6 yr for prostate cancer	Not clear
Nilsson et al., 2015 [12]	Swedish Cancer Registry and nationwide Hospital Discharge Register; Sweden; 1970–2000	Breast cancer	Expected numbers of cerebrovascular events in the background population	Mean age (SD) at time of breast cancer diagnosis = 63.6y (13.9 yr)	Primary outcome = occurrence of stroke (grouped into all stroke, cerebral infarction, cerebral haemorrhage, ill-defined cerebrovascular lesions)	None stated	Excluded	None	25,171 cases	Median follow-up was 5.4 yr (IQR 2.3–10.4 yr)	Not clear
Robinson et al., 2012 [37]	National Prostate Cancer Register (NPCR); Sweden; Nov 2005-Dec 2006. Follow-up to Dec 2007	Prostate cancer	General population of Sweden; matched 1:1 on age (± 1y) and county of residency	< 65–85 + yr no means/medians given, just numbers/%s in each age group category	Primary outcomes = ischaemic heart disease and stroke (although this includes TIA)	Age, number of previous strokes, time since last event, socioeconomic status, and medication for heart disease at baseline	Adjusted for in outcome analysis	None	39,051 cases; 39,051 controls	Cases = mean 1.9 yr (SD 0.4). Controls = mean 2.0 yr (SD 0.4)	0–2 yr
Shin et al., 2018 [38]	Korean National Health Insurance (KNHI) database; South Korea; Jan 2004–Dec 2011. Study follow-up until Dec 2013	Gastric cancer (requiring surgery)	Cancer-free controls propensity score matched 1:1 by year of cancer diagnosis, age, sex, residence, income, disability, hypertension, diabetes, and dyslipidaemia	Mean age (SD) 57.9 yr (± 11.7)	Primary outcomes = coronary heart disease and Ischaemic stroke	Age, sex, insurance premium level, hypertension, diabetes, dyslipidaemia, type of surgery, chemotherapy, smoking status, baseline BMI, and body weight change (decrease defined by > 5% reduction from baseline)	Excluded	2 years	98,936 exposed; 98,936 controls	Cancer population: mean = 5.3 yr. Control population: mean = 5.5 yr	0–10 yr from start of follow-up
Shin et al., 2020 [39]	Korean National Health Insurance (NHIS) database; South Korea; Jan 2007–Dec 2013. Study follow-up until Dec 2016	Prostate cancer	Cancer-free controls matched 1:3 on the basis of age and sex	Cancer population: mean age = 68.4 ± 8.7 yr . Controls: mean = 67.6 ± 9.4 yr	Primary outcomes = newly diagnosed ischaemic heart disease, stroke, or death	Age, income, CCI, hypertension, diabetes, and dyslipidaemia. A screening subset was further adjusted for smoking status, BMI, systolic blood pressure, and total cholesterol	Excluded	1 yr	48,298 cases; 200,480 controls	Cases: mean = 3.65 yr ; controls: mean = 3.85 yr	Not stated
Soisson et al., 2018 [40]	Utah population database; USA; 1997–2012	Endometrial cancer	General population. Matched up to 5:1 on birth year and birth state	< 50 to 90 + yr divided into age groups	Primary outcome = cardiovascular outcomes (hypertension, cerebrovascular diseases, circulatory system disease, heart disease)	Race, baseline BMI, baseline CCI, smoking	Unclear if previous stroke was included or not and no mention on adjustment in analysis if so	1 yr	2648 cases; 10,503 controls	Not stated. Follow-up divided into categories and numbers/% given	1–15 + yr
Strongman et al., 2019 [41]	UK Clinical Practice Research Datalink; UK; 1990–2015	20 adult cancers: oral cavity; oesophageal; stomach; colorectal; liver; pancreas; lung; malignant melanoma; breast; cervix; uterus; ovarian; prostate; kidney; bladder; CNS; thyroid; NHL; multiple myeloma; leukaemia	Controls with no history of cancer. Matched up to 5:1 on age (± 3 yr), sex, and general practice	Cancer survivors: mean = 66.1 yr (SD 13.3) Median = 67 yr (IQR 58–78). Controls: mean = 66.0 yr(13.2) Median = 67 yr (IQR 58–78)	Primary outcomes = risk of cardiovascular disease (coronary artery disease, arrhythmia, heart failure or cardiomyopathy, stroke, PVD, venous thromboembolism, pericarditis, valvular heart diseases)	Age, sex, deprivation, year of cancer diagnosis, smoking, alcohol, BMI, diabetes, hypertension, previous cardiovascular disease, cardiovascular treatments, previous migraine, chronic kidney disease. Additional adjustments for specific cancers: hysterectomy, and HRT (female-specific cancers); chronic liver disease (liver cancer); immunosuppression (NHL); sclerosis and COPD (lung cancer)	Excluded from study	1 yr	108,215 cases; 523,541 controls	Cases = mean 5.7 yr (SD 4.1), median 4.5yr (IQR 2.5–7.9). Controls = mean 6.4 yr (SD 4.2), median 5.4 yr (IQR 3.1–8.8)	Cases = 1–26.5 yr Controls = 1.0–26.6 yr
Suh et al., 2019 [42]	Korean National Health Insurance database (KNHI); South Korea; 2004–2012	Thyroid cancer	General population controls propensity score matched 1:1 on year, age, sex, residence, insurance premium level, disability, hypertension, diabetes, dyslipidaemia	Controls: mean = 47.0 yr (SD 11.3) Cases: mean 47.0 yr (SD 11.3)	Primary outcome = CHD incidence and stroke incidence	-	Excluded	None	182,419 cases; 182,419 controls	Mean follow-up of 4.3 yr	0–10 yr
Tsai et al., 2013 [43]	National Health Insurance Research Database (NHIRD); Taiwan; 2003–2008	Cervical cancer	Patients hospitalised for an appendectomy between 2003 and 2008, matched 2:1 on age	< 45 to 75 + yr. Stratified into groups	Primary outcome = incidence of ischaemic stroke. Secondary outcomes = other vascular events (venous thromboembolism, angina pectoris, MI)	Age, comorbidities, geographic region, urbanisation level, socioeconomic status	Excluded	None	893 cases; 1786 controls	Not stated	0–5 yr
van Hemelrijck et al., 2010 [44]	PCBaSE Sweden, based on the National Prostate Cancer Register (NPCR) of Sweden; Sweden; 1987–2007	Prostate cancer	Swedish male population	< 65 to 75 + yr. Stratified into groups	Primary outcome = cardiovascular disease (ischaemic heart disease, acute MI, arrhythmia, heart failure, stroke)	None	Unclear, stated analysis based on first event after cancer diagnosis	None, not clearly stated	30,642 cases	Mean = 3.5 yr (SD 2.4)	Not stated
van Herk-Sukel et al., 2011 [45]	Dutch National Medical Register of the PHARMO RLS; Netherlands; Jan 2000–Dec 2007	Breast cancer	Cancer-free women selected from all women included in the PHARMO RLS, randomly matched 20:1 by age. 10 matches randomly selected	Mean (SD) 59 yr (± 14)	Primary outcomes = thromboembolic events (MI, ischaemic stroke, PE)	Prior use of antithrombotic drugs, lipid-lowering drugs, antihypertensive drugs, and other cardiovascular drugs	Patients with a previous event (in 12 months prior to cohort entry) excluded from the at-risk population for that specific event	None	11,473 cases; 114,730 controls	Cancer population: mean = 3.7 yr (± 2.2); control population: mean = 3.9 yr (± 2.2)	0–9 yr
van Herk-Sukel et al., 2013 [46]	PALGA, nationwide network and registry of histo- and cytopathology; Netherlands; Jan 2000–Dec 2007	Lung cancer	Cancer-free control from PHARMO RLS, matched randomly 20:1 by age and gender. 10 matches randomly selected	Mean (SD) 66 yr (± 11)	Primary outcomes = thromboembolic events (MI, ischaemic stroke, PE)	Prior use of antithrombotic drugs, antihypertensive drugs	Patients with a previous event (hospitalisation in 12 months before cohort entry) were removed from the at-risk population for that specific event	None	3717 cases; 37,170 controls	SCLC cases, mean = 2.1 yr (± 2.2); NSCLC cases, mean = 2.5 yr (± 2.3); controls, mean = 3.7 yr (± 2.2)	0–9 yr
Wei et al., 2019 [47]	Taiwan National Health Insurance programme; Taiwan; Jan 1996–Dec 2013	Lung, colorectal, hepatocellular carcinoma, urogenital, gastric, prostate, breast, brain, oesophageal, nasopharyngeal, ovarian, thyroid, lymphoma, leukaemia	Non-cancer population Matched 2:1 nested in the same year of cancer diagnosis on age, sex, presence of hypertension, diabetes mellitus, chronic kidney disease, and atrial fibrillation	Cases: mean = 60.6 ± 15.2 yr Controls: mean = 60.6 ± 15.2 yr	Primary outcomes = incidence of stroke	Age, sex, hypertension, diabetes mellitus, chronic kidney disease, atrial fibrillation, coronary artery disease, heart failure, hyperlipidaemia, chronic obstructive pulmonary disease, alcohol overuse	Excluded	None	25,920 cases; 51,840 controls	Not stated	0–1 yr
Wu et al., 2015 [48]	Taiwan National Health Insurance Research Database (NHIRD); Taiwan; cancer diagnosed between 2000 and 2008	Oral cancer	Patients with diseases of the oesophagus, stomach and duodenum and appendicitis. Matched 1:1 by age, gender and index year	Cases: Mean = 52.11 yr (± 11.7); Controls: Mean = 51.91 yr (± 11.88)	Primary outcome = Ischaemic stroke event	Sex, age, CCI, comorbidities	Excluded	None	21,853 cases; 21,853 controls	Not stated	0-10 yr
Zoller et al., 2012 [6]	MigMed 2 database; Sweden; Jan 1987-Dec 2008	All (list in Appendix)	Swedish population without cancer	< 60 to ≥ 80 yr stratified into groups	Primary outcome = Incidence of ischaemic and haemorrhagic stroke	Age (5-year groups), sex, period (5-year groups), region, socioeconomic status, hospitalisation for comorbidities (COPD, obesity, alcoholism, diabetes mellitus, hypertension)	Not stated	None	820,941 cases	Not stated	0–22 yr

*CVD*, cardiovascular disease; *SD*, standard deviation; *yr*, years; *IQR*, inter-quartile range; *CVA*, cerebrovascular accident; *GCC*, germ cell cancer; *BEP*, bleomycin-etoposide-cisplatin; *MTOL*, more than one line of treatment; *MI*, myocardial infarction; *AP*, angina pectoris; *CHF*, congestive heart failure; *ISTH*, International Society on Thrombosis and Haemostasis; *CRNM*, clinically relevant nonmajor; *TIA*, transient ischaemic attack; *CMRN*, Continuous Morbidity Registry Nijmegen; *HF*, heart failure; *SEER*, surveillance, epidemiology, and end results; *CCI*, Charlson comorbidity index; *CNS*, central nervous system; *NHL*, non-Hodgkin’s lymphoma; *PVD*, peripheral vascular disease; *BMI*, body mass index; *CHD*, coronary heart disease; *PE*, pulmonary embolism; *SCLC*, small cell lung cancer; *NSCLC*, non-small cell lung cancer; *COPD*, chronic obstructive pulmonary disease

Eleven of the studies were conducted in Taiwan using the Taiwan National Health Insurance Research Database [19–21, 23, 24, 29, 30, 32, 43, 47, 48] with the remaining studies being conducted in Northern European countries and North America. One study was conducted worldwide in 40 countries [34]. The process yielded a total of 2,226,660 cancer patients.

A variety of cancer types were studied: four prostate [36, 37, 39, 44], three cervical [19, 33, 43], three head and neck [23, 26, 27], three breast [12, 28, 45], two lung [21, 46], two gastric [30, 38], two ovarian [22, 29], two nasopharyngeal [24, 32], and one each of endometrial [40], oral [48], pancreatic [20], Hodgkin’s lymphoma [25], non-Hodgkin’s lymphoma [35], testicular [31], and thyroid [42]. Eight studies evaluated multiple cancer types [4, 6, 16–18, 34, 41, 47]. If not using the general country population as the control group, most studies matched cancer cases with non-cancer cases on age and sex as a minimum.

Studies adjusted for a variety of covariates, the most common being age, sex, and comorbidities. Smoking and cancer treatment received were not consistently adjusted for.

Prior stroke was excluded in 23 studies. Four studies adjusted for prior stroke in their analysis [16, 18, 34, 37]. Nine studies did not state or where not clear on whether they had accounted for prior stroke in analysis [4, 6, 26–28, 32, 33, 40, 44].

Most studies (*n* = 27) did not have any lag period from cancer diagnosis to stroke incidence, and there was a range of duration of follow-up time across the studies.

### Stroke incidence

#### Narrative summary of studies

Across all 36 studies, stroke incidence was expressed using different statistical measures of association, including hazard ratios, incidence rate ratios, standardised incidence ratios, risk ratios, or odds ratios. Several studies reported a combination of association measures. The outcome of stroke incidence according to a categorisation of follow-up time or cancer treatment type was also reported [4, 24, 31, 36, 40, 45, 46].

Of the 28 studies looking at individual cancers and the incidence of stroke, 21 reported an increased incidence of stroke following cancer diagnosis compared to the control group. Seven studies reported a reduced incidence of stroke in individuals with cancer compared to controls (two studies of cervical cancer [19, 33], two of prostate cancer [36, 39], and one each of breast [28], gastric [38], and testicular cancers [31]).

#### Influence of cancer type on stroke incidence

Of eight studies looking at either all cancer types or a specific variety of different cancer types [4, 6, 16–18, 34, 41, 47], the incidence of stroke varied across different cancers. Adelborg et al. investigated haematological cancers and showed that the incidence of stroke was increased in those living with and beyond multiple myeloma, myeloproliferative neoplasms, Hodgkin lymphoma, and myelodysplastic syndrome when compared with the general population [16]. Armenian et al. showed that risk of stroke was increased in those living with and beyond ovarian cancer and lung cancer and was decreased among prostate cancer [17]. The risk of ischaemic stroke was increased for patients with lung, colon, bladder, rectal, or pancreatic cancer in Andersen et al., while haemorrhagic stroke risk was only significantly increased in patients with lung cancer [18].

Navi et al. looked at patients with a diagnosis of breast, colorectal, lung, pancreatic, or prostate cancer; cumulative incidence rates of stroke were higher in all cancer groups compared with matched controls. These were most pronounced for lung, pancreatic, and colorectal cancer patients [4]. Strongman et al. showed increased risk of stroke in eight out of 20 cancers compared to controls from the general population. These were oral, pancreas, lung, cervical, CNS, non-Hodgkin’s lymphoma, multiple myeloma, and leukaemia [41]. Wei et al. combined a range of cancers (colorectal, lung, urogenital, gastric, prostate, breast, brain, oesophageal, nasopharyngeal, ovarian, thyroid, hepatocellular carcinoma, leukaemia, and lymphoma) and showed that there was an increased risk of stroke within the year after cancer diagnosis [47]. Risk of ischaemic stroke was increased after cancer diagnosis in 20 of 34 cancers studied by Zoller et al. [6]. This risk was more than two times greater for small intestine, pancreas, lung, nervous system, endocrine glands, and leukaemia. The risk of haemorrhagic stroke was increased in 18 of the 34 cancers studied. This was greatest for small intestine, liver, kidney, nervous system, thyroid gland, endocrine glands, connective tissue, non-Hodgkin lymphoma, myeloma, and leukaemia. The only RCT included found no association between cancer cases and non-cancer cases and the incidence of stroke [34].

#### Influence of cancer treatment on stroke incidence

Twenty-one studies investigated cancer treatment and its association with incidence of stroke (Supplementary Table 2). Stroke risk was highest in patients receiving both radiotherapy and chemotherapy in head and neck cancer [23] compared with no treatment or singular treatment. Patients treated with neck irradiation together with mediastinal irradiation experienced a statistically significant increased risk for ischemic stroke compared with those treated with chemotherapy or infradiaphragmatic irradiation alone in a study of Hodgkin lymphoma survivors [25]. Non-Hodgkin lymphoma survivors treated with chemotherapy also had increased incidence of stroke compared to the general population [41]. Ischaemic stroke incidence rates were significantly higher in nasopharyngeal cancer patients after radiotherapy, radiotherapy/chemotherapy, and non-radio/chemotherapy than in the reference control population. Those receiving radiotherapy/chemotherapy had the highest incidence followed by those receiving radiotherapy only [24]. Incidence of stroke was increased in lung cancer survivors following singular treatment with radiotherapy or chemotherapy in addition to both chemotherapy and radiotherapy [41]. Cisplatin-based and carboplatin-based chemotherapy regimes for ovarian cancer were independent risk factors for ischaemic stroke, whereas non-platinum-based regimens were not [29]. Those within a palliative treatment regimen for prostate cancer had increased risk for ischaemic stroke [36], whereas stroke incidence was higher in those treated with endocrine therapy for prostate cancer [39, 44]. In one study for prostate cancer, only gonadotrophin-releasing hormone agonists resulted in an increased incidence of stroke [37]. In oral cancer patients, stroke incidence was highest in those treated with radiotherapy/chemotherapy/concurrent chemoradiotherapy compared to those treated with surgery alone [48]. Risk of stroke did not differ between breast cancer patients who were treated with surgery alone and those who received radiotherapy in combination with surgery. However, among patients who were treated with radiotherapy plus hormone therapy, an elevated risk of stroke was observed compared with the general population [28]. For patients with testicular cancer, risks for stroke were investigated in patients undergoing surveillance, radiotherapy, or bleomycin-etoposide-cisplatin (BEP) treatment. Only patients receiving BEP had an increased risk of cerebrovascular accident incidence in the year following treatment [31].

#### Influence of time since cancer diagnosis and stroke incidence

Follow-up time was categorised in seventeen studies in relation to the outcome of stroke incidence (Supplementary Table 3). For breast, colorectal, lung, pancreatic, and prostate cancer, stroke risk was greatest in the first month following cancer diagnosis, and that risk attenuated over time and was generally no longer present beyond 1 year in one study [4]. In other studies, this risk remained elevated for up to 10 years following cancer diagnosis [6, 12, 20, 41]. Zoller et al. showed that the standardised incidence ratios (SIR) for ischaemic stroke and haemorrhagic stroke increased during the first 6 months after diagnosis in 23 and 15 of 34 cancers, respectively [6]. It decreased thereafter but remained relatively constant after 6 months. A high incidence of both ischaemic and haemorrhagic stroke was observed within 6 months after diagnosis in a pancreatic cancer cohort; this declined with time and was no longer significant 24 months after the cancer diagnosis [20]. Risk of stroke for those with lung cancer was highest during the first 3 months for men and within 4 to 6 months for women and, then, decreasing over 1 year of follow-up for men and 2 years of follow-up for women [21]. Incidence rates for stroke in women with ovarian cancer were over two times higher than cancer-free women 3 months after cancer diagnosis and decreased but still elevated by 12 months [22].

People living with and beyond head and neck cancers had a consistently elevated stroke incidence over 8 years from cancer diagnosis [23, 26]. Hodgkin lymphoma patients experienced an approximately twofold increased SIR for stroke risk compared to the general population with no increasing or decreasing trends with longer follow-up time [25]. Non-Hodgkin lymphoma patients also had increased incidence of stroke which continued for over 10 years following diagnosis [6, 16, 41]. Increased stroke risk was seen in patients receiving BEP treatment for testicular cancer in the year following diagnosis; however, this risk decreased and was no longer significant after 1 year [31]. Nilsson et al. observed a 22% increase in the risk of stroke in the first year after breast cancer diagnosis. By 1 to 5 years, there was no significant increase, but then, an increase was observed again after 5 to 10 years of follow-up and after greater than 10 years of follow-up [12]. Soisson et al. followed patients from 1 year after endometrial cancer diagnosis, and there was no significantly increased risk for stroke between 1 to 10 years from cancer diagnosis [40]. In breast cancer patients, stroke incidence was increased, but this did not reach significance over three time periods (0 to 6 month, 6 to 12 month, or 12-month to total follow-up) from diagnosis [45]. In another study by van Herk-Sukel et al., there was a trend towards increased incidence in the 0 to 6 months following lung cancer diagnosis [46].

#### Influence of smoking and stroke incidence in individuals living with and beyond cancer

Smoking status and incidence of stroke in individuals living with and beyond cancer in comparison to the matched general population cohort were investigated in three studies [26, 35, 41] (Supplementary Table 4). Strongman et al. showed an increased incidence of stroke in survivors with non-Hodgkin lymphoma, lung, breast, or leukaemia who had ever smoked compared with the general population cohort [41]. No association was observed in survivors with colorectal, malignant melanoma, uterine, prostate, or bladder cancer [41]. Dorresteijn et al. observed an increased stroke risk in both non-smokers and smokers living with and beyond head and neck cancer in comparison with the general population [26]. Smoking status had no association with an increased incidence of stroke in those with non-Hodgkin lymphoma [35].

The association between cancers related to smoking and increased stroke risk was investigated in two studies [6, 18] (Supplementary Table 4). Andersen et al. grouped cancers into those strongly associated with smoking (lung, colon, bladder, rectum, pancreas, kidney, stomach, and head and neck) and those less strongly associated with smoking (non-Hodgkin lymphoma, breast, prostate, melanoma, CNS, ovary, and endometrial). They found that the risk of ischemic and haemorrhagic stroke was increased for all smoking-related cancers combined but not non-smoking-related cancers [18]. In another study, several non-smoking-related cancers were associated with an increased risk of haemorrhagic and ischaemic strokes [6]; these included small intestine, colon, rectum, breast, endometrium, ovary, other female genital, prostate, melanoma, nervous system, endocrine glands, connective tissue, non-Hodgkin lymphoma, and myeloma. This risk declined rapidly after 6 months but remained raised for 10 or more years [6].

Several other studies within the review looked at smoking and increased stroke risk but only within the cohort of cancer patients [25, 28, 38, 42] (Supplementary Table 4). Only one of these studies into thyroid cancer found an increased risk of stroke incidence in current smokers [42]; the others found no association.

#### Meta-analysis

**Fig. 2:**
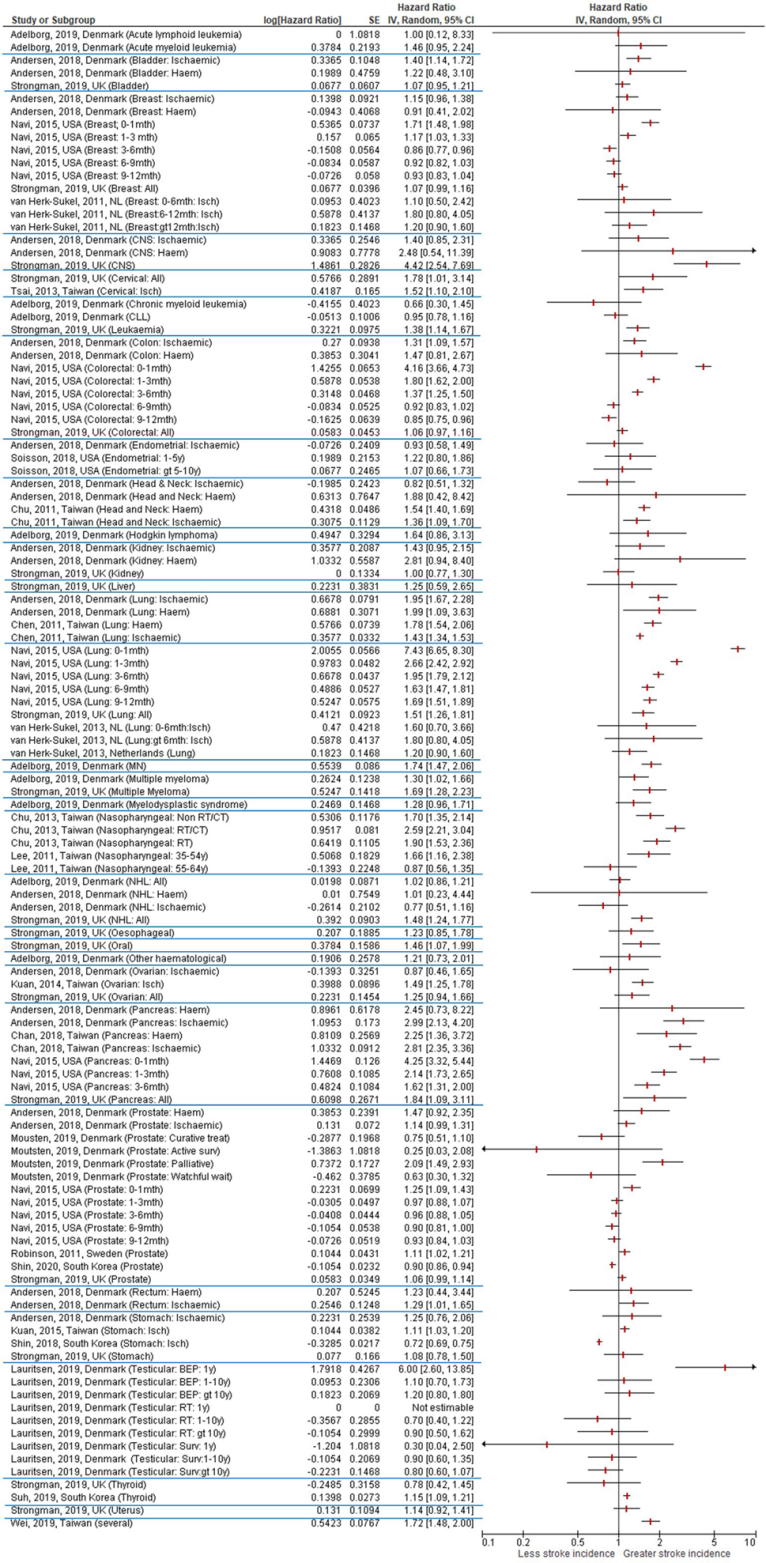
Hazard ratio for stroke incidence with cancer (*blue lines show distinction between different cancer types*) larger font for fig 2 and Fig 3 , font needs be increased so reader can see info presented

**Fig. 3:**
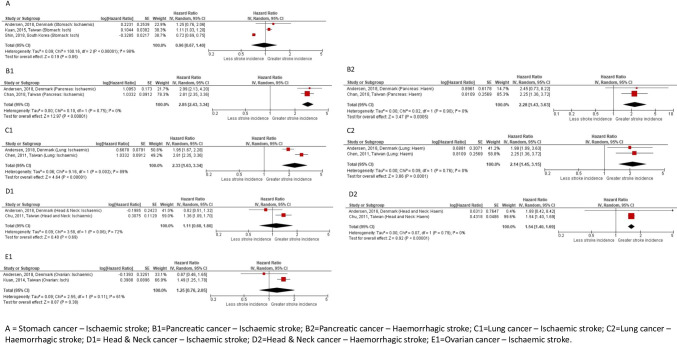
Meta-analysis: Hazard ratio for stroke incidence in survivors of adult cancers.

Twenty-five of the included studies calculated hazard ratios for the incidence of stroke. The HR estimates for stroke incidence in all cancer types ranged from 0.25 to 7.43 (Table 2 and Fig. 2). Seven studies were included in the meta-analysis for individual cancer types (lung, pancreatic, stomach, ovarian, head and neck) (Fig. 3). Overall pooled HR of ischaemic stroke incidence was significant for lung cancer (HR 2.33, 95% CI 1.63–3.34) and pancreatic cancer (HR 2.85, 95% CI 2.43–3.34). For haemorrhagic stroke, the overall pooled HR remained significant for lung cancer (HR 2.14, 95% CI 1.45–3.15), pancreatic cancer (HR 2.28, 95% CI 1.43–3.63) and was also significant for head and neck cancer (HR 1.54 (95% CI 1.40–1.69). Pooled HR were not significant for either ischaemic stroke only for stomach cancer, head and neck cancer, and ovarian cancer (Fig. 3). Between-study variation was high for stomach cancer, lung cancer, head and neck cancer, and ovarian cancer when looking at ischaemic stroke.

**Table 2:** Incidence of stroke in studies using hazard ratio

Study (author, year, reference)	Control group	Cancer group	Control group incidence rate	Cancer group incidence rate	Time period	Unadjusted HR (95% CI) (*p* value)	Adjusted HR (95% CI) (*p* value)
**Adelborg et al., 2019 **[16] *(haematological cancers)*							
*All haem cancers*							1.22 (1.12–1.33)
*Hodgkin lymphoma*	55/27133 person years	13/4795 person years	2.03 (1.56–2.64)	2.71 (1.58–4.67)			1.64 (0.86–3.13)
*Non-Hodgkin lymphoma*	1187/205,729 person years	172/31,464 person years	5.77 (5.45–6.11)	5.47 (4.71–6.35)			1.02 (0.86–1.21)
*Acute myeloid leukaemia*	481/74,859 person years	28/4880 person years	6.43 (5.88–7.03)	5.74 (3.96–8.31)			1.46 (0.95–2.24)
*Acute lymphoid leukaemia*	28/10,565 person years	1/1171 person years	2.65 (1.833.84)	0.85 (0.12–6.04)			1.00 (0.12–6.04)
*Chronic myeloid leukaemia*	133/23,741 person years	7/3379 person years	5.60 (4.73–6.64)	2.07 (0.99–4.35)			0.66 (0.30–1.47)
*Chronic lymphocytic leukaemia*	937/123,732 person years	129/20,801 person years	7.57 (7.10–8.07)	6.20 (5.22–7.37)			0.95 (0.78–1.15)
*Multiple myeloma*	735/107,567 person years	88/11,895 person years	6.83 (6.36–7.35)	7.40 (6.01–9.12)			1.30 (1.02–1.65)
*Myeloproliferative neoplasms*	740/105,579 person years	209/18,629 person years	7.01 (6.52–7.53)	11/22 (9.80–12.85)			1.74 (1.47–2.04)
*Myelodysplastic syndrome*	536/58,999 person years	64/6649 person years	9.09 (8.35–9.89)	9.63 (7.54–12.31)			1.28 (0.96–1.69)
*Other haematological cancers*	170/26,108 person years	20/3514 person years	6.51 (5.60–7.57)	5.70 (3.68–8.83)			1.21 (0.73–1.98)
**Andersen et al., 2018 **[18] * (also stratified by cancer type and non-smoking cancers)							
*All stroke*	9659/2514534	1078/261404					
*Ischaemic stroke*	8817/2514534	984/261404					1.3 (1.23–1.38)
*Haemorrhagic stroke*	842/2514534	94/261404					1.41 (1.14–1.75)
*Bladder –ischaemic*	-	-					1.4 (1.14–1.72)
*Colon – ischaemic*	-	-					1.31 (1.09–1.56)
*Head and neck – ischaemic*	-	-					0.82 (0.51–1.32)
*Kidney – ischaemic*	-	-					1.43 (0.95–2.15)
*Lung – ischaemic*	-	-					1.95 (1.67–2.28)
*Pancreas – ischaemic*	-	-					2.99 (2.13–4.19)
*Rectum – ischaemic*	-	-					1.29 (1.01–1.65)
*Stomach – ischaemic*	-	-					1.25 (0.76–2.04)
*Breast – ischaemic*	-	-					1.15 (0.96–1.37)
*Endometrial – ischaemic*	-	-					0.93 (0.58–1.47)
*Non-Hodgkin – ischaemic*	-	-					0.77 (0.51–1.17)
*Ovarian – ischaemic*	-	-					0.87 (0.46–1.66)
*Prostate – ischaemic*	-	-					1.14 (0.99–1.30)
*CNS—ischaemic*	-	-					1.4 (0.85–2.3)
*Bladder – haemorrhagic*	-	-					1.22 (0.48–3.08)
*Colon – haemorrhagic*	-	-					1.47 (0.81–2.68)
*Head and neck – haemorrhagic*	-	-					1.88 (0.42–8.44)
*Kidney – haemorrhagic*	-	-					2.81 (0.94–8.37)
*Lung – haemorrhagic*	-	-					1.99 (1.09–3.63)
*Pancreas – haemorrhagic*	-	-					2.45 (0.73–8.20)
*Rectum – haemorrhagic*	-	-					1.23 (0.44–3.47)
*Breast – haemorrhagic*	-	-					0.91 (0.41–1.96)
*Non-Hodgkin – haemorrhagic*	-	-					1.01 (0.23–4.30)
*Prostate – haemorrhagic*	-	-					1.47 (0.92–2.36)
*CNS – haemorrhagic*	-	-					2.48 (0.54–11.28)
**Chang et al., 2013 **[19] *(cervical cancer)*	8375/415991 person years (*n* = 81,144)	1187/94592 person years (*n* = 20,286)	20.1 (per 1000 person years)	12.5 (per 1000 person years)		0.62 (0.58–0.66) (*p* < 0.0001)	0.58 (0.54–0.61) (*p* < 0.0001)
**Chan et al., 2018 **[20] *(pancreatic cancer)*							
*All stroke*	1000/29916	172/7479	12.3 (per 1000 person years)	28.5 (per 1000 person years)		2.23 (1.88–2.63) (*p* < 0.001)	2.74 (2.31–3.24) (*p* < 0.001)
*Ischaemic stroke*	870/29916	153/7479	10.7 (per 1000 person years)	25.4 (per 1000 person years)		2.26 (1.90–2.70) (*p* < 0.001)	2.81 (2.35–3.37) (*p* < 0.001)
*Haemorrhagic stroke*	130/29916	19/7479	1.60 (per 1000 person years)	3.15 (per 1000 person years)		1.96 (1.19–3.21) (*p* < 0.01)	2.25 (1.36–3.71) (*p* < 0.01)
**Chen et al., 2011 **[21] *(lung cancer)*							
*All strokes*	8172/104178	1728/52089	17.43 (per 1000 person years)	25.87 (per 1000 person years)		1.42 (1.35–1.50)	1.47 (1.39–1.56)
*Ischaemic stroke*	7079/104178	1456/52089	15.10 (per 1000 person years)	21.80 (per 1000 person years)		1.38 (1.30–1.46)	1.43 (1.34–1.51)
*Haemorrhagic stroke*	1093/104178	272/52089	2.33 (per 1000 person years)	4.07 (per 1000 person years)		1.69 (1.47–1.94)	1.78 (1.54–2.05)
**Chu et al., 2011 **[23] *(head & neck cancer)*							
*All strokes*	2915/53517	694/13390	7.9 (per 1000 person years)	11.4 (per 1000 person years)			1.52 (1.40–1.65) (*p* < 0.0001)
*Ischaemic stroke*	2460/53517	596/13390	6.67 (per 1000 person years)	9.77 (per 1000 person years)		1.32 (1.06–1.64) (*p* < 0.05)	1.36 (1.09–1.69) (*p* < 0.05)
*Haemorrhagic stroke*	455/53517	98/13390	1.23 (per 1000 person years)	1.61 (per 1000 person years)		1.47 (1.34–1.60) (*p* < 0.0001)	1.54 (1.40–1.68) (*p* < 0.0001)
**Chu et al., 2013 **[24] *(nasopharyngeal cancer)*							
*Radiotherapy*	1866/307,232 person years	102/7418 person years	6.07	13.75			1.90 (1.53–2.35)
*Radiotherapy/chemotherapy*	1866/307,232 person years	179/14030 person years	6.07	12.76			2.59 (2.21–3.03)
*Non-radiotherapy/chemotherapy*	1866/307,232 person years	90/6076 person years	6.07	13.17			1.70 (1.35–2.15)
**Kuan et al., 2014 **[29] *(ovarian cancer)*	244/8810	267/8810	6.8 (per 1000 person years)	9.4 (per 1000 person years)		1.38 (1.16–1.64) (*p* < 0.001)	1.49 (1.25–1.78) (*p* < 0.001)
**Kuan et al., 2015 **[30] *(gastric cancer)*	1893/22530	1106/22530	21.4 (per 1000 person years)	22.6 (per 1000 person years)		1.05 (0.97–1.13) (*p* = 0.215)	1.11 (1.03–1.19) (*p* = 0.007)
**Lauritsen et al., 2019 **[31] *(testicular cancer and GCC of extragonadal origin)*							
*Surveillance*	Events per person years	Events per person years					
< *1-year follow-up*	47/51246	< 4/3021					0.3 (0.0–2.5)
*1–10-year follow-up*	528/419366	23/20714					0.9 (0.6–1.3)
> *10-year follow-up*	964/347895	40/16582					0.8 (0.6–1.2)
*BEP (bleomycin-etoposide-cisplatin)*							
< *1-year follow-up*	47/51246	6/1379					6.0 (2.6–14.1)
*1–10-year follow-up*	528/419366	14/12653					1.1 (0.7–1.9)
> *10-year follow-up*	964/347895	27/10507					1.2 (0.8–1.7)
*Radiotherapy*							
< *1-year follow-up*	47/51246	0/578					-
*1–10-year follow-up*	528/419366	7/5691					0.7 (0.4–1.6)
> *10-year follow-up*	964/347895	14/4482					0.9 (0.5–1.5)
**Lee et al., 2011 **[32] *(nasopharyngeal cancer)*							
*Patients aged 35–54*	101/3108	37/777	3.2%	4.8%		1.70 (1.16–2.47) *p* = 0.006)	1.66 (1.16–2.86) (*p* = 0.009)
*Patients aged 55–64*	129/1268	25/317	10.2%	7.9%		0.91 (0.59–1.40) (*p* = 0.661)	0.87 (0.56–1.33) (*p* = 0.524)
**Melloni et al., 2017 **[34] *(any cancer, excluding basal or squamous cell skin cancer)*							
*Stroke or SE*	447/16947	29/1236	1.4%	1.3%		0.89 (0.61–1.30) (*p* = 0.5575)	0.93 (0.63–1.37) (*p* = 0.7104)
*Ischaemic stroke*	313/16947	23/1236	1.0%	1.0%		1.01 (0.66–1.55) (*p* = 0.9559)	1.02 (0.66–1.58) (*p* = 0.9179)
**Moutsten et al., 2019 **[36] *(prostate cancer)*							
*Active surveillance*	2144/25436	< 5/133					0.25 (0.03–1.76)
*Watchful waiting*	2144/25436	< 10/230					0.63 (0.30–1.33)
*Curative intended treatment*	2144/25436	25/695					0.75 (0.51–1.12)
*Palliative treatment*	2144/25436	35/488					2.09 (1.49–2.93)
**Navi et al., 2015 **[4]							
*Breast cancer*							
*3 months since diagnosis*			1.1% (95% CI 1.0–1.2)	1.5% (95% CI 1.4–1.6)	*0*–*1 months after diagnosis*	1.71 (1.48–1.99)	
*6 months since diagnosis*			2.1% (95% CI 2.0–2.2)	2.3% (95% CI 2.2–2.4)	*1*–*3 months after diagnosis*	1.17 (1.03–1.32)	
*1 year since diagnosis*			3.9% (95% CI 3.7–4.0)	3.9% (95% CI 3.8–4.1)	*3*–*6 months after diagnosis*	0.86 (0.77–0.96)	
*2 years since diagnosis*			6.7% (95% CI 6.5–6.9)	6.3% (95% CI 6.2–6.5)	*6*–*9 months after diagnosis*	0.92 (0.82–1.04)	
*3 years since diagnosis*			8.9% (95% CI 8.7–9.1)	8.4% (95% CI 8.1–8.6)	*9*–*12 months after diagnosis*	0.93 (0.83–1.04)	
*Colorectal cancer*							
*3 months since diagnosis*			1.3% (95% CI 1.2–1.4)	3.3% (95% CI 3.2–3.4)	*0*–*1 months after diagnosis*	4.16 (3.66–4.72)	
*6 months since diagnosis*			2.4% (95% CI 2.3–2.6)	4.7% (95% CI 4.5–4.8)	*1*–*3 months after diagnosis*	1.80 (1.62–2.00)	
*1 year since diagnosis*			4.6% (95% CI 4.4–4.7)	6.2% (95% CI 6.0–6.4)	*3*–*6 months after diagnosis*	1.37 (1.25–1.51)	
*2 years since diagnosis*			8.0% (95% CI 7.8–8.2)	8.4% (95% CI 8.2–8.7)	*6*–*9 months after diagnosis*	0.92 (0.83–1.03)	
*3 years since diagnosis*			10.4% (95% CI 10.2–10.7)	10.1% (95% CI 9.9–10.3)	*9*–*12 months after diagnosis*	0.85 (0.75–0.95)	
*Lung cancer*							
*3 months since diagnosis*			1.2% (95% CI 1.2–1.3)	5.1% (95% CI 4.9–5.2)	*0*–*1 months after diagnosis*	7.43 (6.65–8.29)	
*6 months since diagnosis*			2.4% (95%CI 2.3–2.5)	6.6% (95% CI 6.4–6.7)	*1*–*3 months after diagnosis*	2.66 (2.42–2.91)	
*1 year since diagnosis*			4.4% (95% CI 4.2–4.5)	8.1% (95% CI 8.0–8.3)	*3*–*6 months after diagnosis*	1.95 (1.79–2.12)	
					*6*–*9 months after diagnosis*	1.63 (1.47–1.80)	
					*9*–*12 months after diagnosis*	1.69 (1.51–1.88)	
*Pancreatic cancer*							
*3 months since diagnosis*			1.3% (95% CI 1.1–1.5)	3.4% (95% CI 3.1–3.6)	*0*–*1 months after diagnosis*	4.25 (3.32–5.45)	
*6 months since diagnosis*			2.3% (95% CI 2.1–2.6)	4.3% (95% CI 4.0–4.6)	*1*–*3 months after diagnosis*	2.14 (1.73–2.65)	
					*3*–*6 months after diagnosis*	1.62 (1.31–2.01)	
*Prostate cancer*							
*3 months since diagnosis*			1.1% (95% CI 1.0–1.2)	1.2% (95% CI 1.1–1.3)	*0*–*1 months after diagnosis*	1.25 (1.09–1.43)	
*6 months since diagnosis*			2.1% (95% CI 2.0–2.2)	2.1% (95% CI 2.0–2.2)	*1*–*3 months after diagnosis*	0.97 (0.88–1.08)	
*1 year since diagnosis*			3.8% (95% CI 3.6–3.9)	3.6% (95% CI 3.5–3.8)	*3*–*6 months after diagnosis*	0.96 (0.88–1.05)	
*2 years since diagnosis*			6.5% (95% CI 6.4–6.7)	6.3% (95% CI 6.2–6.5)	*6*–*9 months after diagnosis*	0.90 (0.81–0.99)	
*3 years since diagnosis*			8.7% (95% CI 8.5–8.8)	8.3% (95% CI 8.1–8.5)	*9*–*12 months after diagnosis*	0.93 (0.84–1.03)	
**Robinson et al., 2011 **[37] *(prostate cancer)*	1006/39051	1105/39051	13.3 (per 1000 person years)	14.8 (per 1000 person years)			1.11 (1.02–1.21)
**Shin et al., 2018 **[38] *(gastric cancer)*	5979/98936	4849/98936					0.72 (0.69–0.75)
**Shin et al., 2020 **[39] *(prostate cancer)*							
*All participants*	9028/200480	1980/48298	12.0 (per 1000 person years)	11.5 (per 1000 person years)			0.90 (0.86–0.95)
*Screening subset*	4002/105347	1075/29365	10.4 (per 1000 person years)	10.4 (per 1000 person years)			0.98 (0.91–1.05)
**Soisson et al., 2018 **[40] *(endometrial cancer)*							
*1*–*5 y after cancer diagnosis*	212/10503	56/2648					1.22 (0.80–1.87)
> *5–10 y after cancer diagnosis*	203/10503	47/2648					1.07 (0.66–1.74)
**Strongman et al., 2019 **[41]							
*Oral cavity cancer*			8.1	11.2			1.46 (1.07–2.00)
*Oesophageal cancer*			11.6	11.2			1.23 (0.85–1.79)
*Stomach cancer*			13.8	14.2			1.08 (0.78–1.49)
*Colorectal cancer*			12.0	12.3			1.06 (0.97–1.17)
*Liver cancer*			9.8	13.4			1.25 (0.59–2.64)
*Pancreatic cancer*			10.7	15.4			1.84 (1.09–3.12)
*Lung cancer*			11.6	19.5			1.51 (1.26–1.82)
*Breast cancer*			6.6	6.8			1.07 (0.99–1.16)
*Cervical cancer*			3.4	4.1			1.78 (1.01–3.17)
*Uterine cancer*			7.6	8.3			1.14 (0.92–1.42)
*Ovarian cancer*			6.3	7.2			1.25 (0.94–1.66)
*Prostate cancer*			14.3	14.5			1.06 (0.99–1.14)
*Kidney cancer*			10.0	11.0			1.00 (0.77–1.30)
*Bladder cancer*			13.6	15.1			1.07 (0.95–1.19)
*CNS cancer*			4.2	13.2			4.42 (2.54–7.72)
*Thyroid cancer*			4.1	3.8			0.78 (0.42–1.42)
*Non-Hodgkin’s lymphoma*			8.9	11.2			1.48 (1.24–1.76)
*Multiple myeloma*			11.1	16.3			1.69 (1.28–2.23)
*Leukaemia*			10.7	13.0			1.38 (1.14–1.67)
**Suh et al., 2019 **[42] *(thyroid cancer)*	2550/182419	2914/182419		3.71 per 1000 person years			1.15 (1.09–1.22)
**Tsai et al., 2013 **[43] *(cervical cancer)*	91/1786	70/893	5.1%	7.8%		1.56 (1.14–2.13) (0.005)	1.52 (1.10–2.08) (*p* = 0.01)
**van Herk-Sukel et al., 2011 **[45] *(breast cancer)*							
*0 to 6 months after breast cancer hospitalisation*	46 (follow-up person years 55,580)	8 (follow-up person years 5505)	0.8 (95% CI 0.6–1.1) (per 1000 person years)	1.5 (95% CI 0.5–2.9) per 1000 person years		1.8 (0.8–3.8)	1.1 (0.5–2.5)
*6–12 months after breast cancer hospitalisation*	39 (follow-up person years 51,730)	9 (follow-up person years 5089)	0.8 (95% CI 0.5–1.0) per 1000 person years	1.8 (95% CI 0.8–3.3) per 1000 person years		2.4 (1.1–4.9)	1.8 (0.8–3.9)
*12 months to total follow-up after breast cancer hospitalisation*	349 (follow-up person years 334,624)	50 (follow-up person years 31,438)	1.0 (95% CI 0.9–1.2) per 1000 person years	1.6 (95% CI 1.2–2.1) per 1000 person years		1.5 (1.1–2.1)	1.2 (0.9–1.6)
**van Herk-Sukel et al., 2013 **[46] *(lung cancer)*							
*0–6 months after lung cancer diagnosis*	37	6	2.1 (95% CI 1.4–2.8) per 1000 person years	3.8 (95% CI 1.3–8.3) per 1000 person years		2.0 (0.8–4.8)	1.6 (0.7–4.0)
*6 months after lung cancer diagnosis to total follow-up*	235	13	2.0 (95% CI 1.8–2.3) per 1000 person years	1.8 (95% CI 1.0–3.0) per 1000 person years		0.9 (0.5–1.6)	0.7 (0.4–1.3)
**Wei et al., 2019 **[47] (several cancers)	-	-					1.72 (1.48–2.01) (*p* < 0.0001)
**Wu et al., 2015 **[48] *(oral cancer)*	409/21853	1514/21853	3.82 (per 1000 person years)	20.56 (per 1000 person years)		Cancer group as reference	Cancer group as reference
						Control group 0.26 (0.23-0.29) (<0.0001)	Control group 0.23 (0.21-0.26) (<0.0001)

^*^RR presented by means of HR by cox regression

*HR*, hazard ratio; *CI*, confidence interval; *GCC*, germ cell cancer; *yr* = years; *CNS* = central nervous systems; *SE* = systemic embolism

In the remaining 11 studies, other measures of stroke incidence were used. Supplementary Table 5 shows the incidence rate ratio for stroke in four studies, where most cancer types within the studies showed an increased rate ratio. Six studies (Supplementary Table 6 ) calculated SIR, two of the studies reported SIRs of less than 1, whereas the remaining studies had SIRs of greater than 1. Two studies (Supplementary Table 7 ) reported odds ratios which were greater than 1; however, the 95% confidence interval included 1. Pooled ratios were not possible due to there being only one estimate per cancer type across all of the studies.

#### Quality assessment/risk of bias

Median quality score was 7 with a range of 5 to 9 (Table 3). There were five studies with a score of 5 or 6; however none of these formed part of the meta-analysis as they did not report hazard ratios. The low scores were mainly due to inadequacy of the follow-up period of the patient cohorts, lack of adjustment for additional factors in the comparability of the patient cohorts, and no demonstration that participants had not had a stroke prior to the start of the study. Only five out of the 36 studies were adjusted for smoking in the analysis which may be an important risk factor for stroke incidence.

**Table 3:** Quality assessment of included studies

Study	Selection (maximum of 4 stars)	Comparability (maximum of 2 stars)	Exposure (maximum of 3 stars)	Total (maximum of 9)
Adelborg et al., 2019 [16]	****	*	**	7
Armenian et al., 2016 [17]	****	**	**	8
Andersen et al., 2018 [18]	****	**	***	9
Chang et al., 2013 [19]	****	*	**	7
Chan et al., 2018 [20]	****	*	**	7
Chen et al., 2011 [21]	****	*	**	7
Chia et al., 2013 [22]	****	*	***	8
Chu et al., 2011 [23]	****	*	**	7
Chu et al., 2013 [24]	****	*	**	7
De Bruin et al., 2009 [25]	***	**	**	7
Dorresteijn et al., 2002 [26]	*	**	**	5
Haynes et al.^2^, 2002 [27]	***	**	**	7
Hooning et al., 2006 [28]	**	**	**	6
Kuan et al., 2014 [29]	****	*	**	7
Kuan et al., 2015 [30]	****	*	**	7
Lauritsen et al., 2019 [31]	****	*	**	7
Lee et al., 2011 [32]	***	*	**	6
Maduro et al., 2010 [33]	**	*	**	5
Moser et al., 2006 [35]	***	**	***	8
Moutsten et al., 2019 [36]	****	**	***	9
Navi et al., 2015 [4]	***	*	***	7
Nilsson et al., 2015 [12]	****	*	***	8
Robinson et al., 2011 [37]	***	*	***	7
Shin et al., 2018 [38]	****	**	***	9
Shin et al., 2020 [39]	****	**	***	9
Soisson et al., 2018 [40]	****	**	***	9
Strongman et al., 2019 [41]	****	**	***	9
Suh et al., 2019 [42]	****	**	**	8
Tsai et al., 2013 [43]	****	*	**	7
van Hemelrijck et al., 2010 [44]	**	*	**	5
van Herk-Sukel et al., 2011 [45]	****	*	**	7
van Herk-Sukel et al., 2013 [46]	****	*	**	7
Wei et al., 2019 [47]	****	**	**	8
Wu et al., 2015 [48]	****	*	**	7
Zoller et al., 2012 [6]	****	*	***	8

A comparison of scorings was undertaken, and the kappa statistic was in strong agreement of 0.61.

## Discussion

### Principal findings

Meta-analysis of observational studies shows that individuals living with and beyond cancer had a higher incidence of stroke than demographically similar individuals who have not been diagnosed with cancer. The risk of ischaemic stroke is significantly higher in those with lung cancer (2 studies, HR 2.33, 95% CI 1.63–3.35), pancreatic cancer (2 studies, HR 2.85, 95% CI 2.43–3.36), and haemorrhagic stroke in those with lung cancer (2 studies, HR 2.14, 95% CI 1.45–3.15), pancreatic cancer (2 studies, HR 2.28, 95% CI 1.43–3.63), and head and neck cancer (2 studies, HR 1.54, 95% CI 1.40–1.69) compared to controls. No statistically significant increase in stroke incidence was observed in individuals with stomach cancer or ovarian cancer. In the narrative synthesis, several studies also showed significantly increased incidence of stroke in individuals with colorectal cancer, breast cancer, ovarian cancer, nasopharyngeal cancer, thyroid cancer, leukaemia, and myeloma [4, 6, 16, 18, 24, 25, 29, 32, 41, 42]. Narrative synthesis identified that the relationship between stroke incidence and cancer is not a simple one. Cancer type, time since diagnosis of cancer, the types of anti-cancer treatments received, and shared risk factors such as smoking can all influence the risk of stroke after cancer.

### Comparison with existing literature

Earlier reviews have also noted increased incidence of stroke in cancer patients. A recent meta-analysis has shown that the overall relative risk for stroke in cancer patients was 1.66 (95% CI 1.35–2.04) [54]. This study included childhood cancers and pooled effects of association together, including SMR, and only identified 20 cohort studies as opposed to our 35 observational studies and one RCT. Previous reviews have also hinted that treatment type is important, with increased incidence of stroke noted in individuals receiving androgen deprivation therapy for prostate cancer, tamoxifen treatment for breast cancer, and radiotherapy in several different cancers [49–52]. The time between cancer diagnosis and incident stroke was discussed by Navi et al. in a narrative review of arterial thromboembolism and cancer [53]. The authors hypothesised that a “U”-shaped curve might exist, in which stroke risk is highest soon after diagnosis due to cancer-mediated hypercoagulability and then decreases as cancer is controlled by treatment, before increasing again in the long-term due to late effects of cancer treatment (particularly radiotherapy).

### Potential mechanisms of increased stroke incidence after cancer

Several mechanisms, alone or together, may account for the increased risk of stroke in patients with cancer. Stroke risk has been shown to directly correlate with cancer stage, with stage 4 cancers demonstrating the highest risks [4, 6] including a more than tenfold increased risk in the first month after cancer diagnosis [5] indicating that both tumour burden (tumour emboli, vessel compression, or infiltration) and cancer-associated coagulopathy can underlie or enhance the occurrence of stroke in cancer patients. In our meta-analysis, lung and pancreatic cancer, which are known to be diagnosed at a later stage [55], showed significantly increased risk of stroke. Brain imaging studies have revealed that cancer patients with stroke often exhibit multiple arterial territorial involvements, suggesting an embolic origin, compared with the single infarct seen in those with conventional stroke risks [56].

Hypercoagulability associated with tumour proliferation may also contribute to the increased risk of stroke in cancer patients [25]. Follow-up times varied between the studies in this review; however, stroke risk among cancer survivors has been shown to be highest soon after diagnosis [12, 18] and declines over time [4, 6, 20, 21, 46]. This perhaps reflects a decreased tumour burden after treatment intervention, cessation of cytostatic agents (decreased chemotherapy-related complications), or death-related selective bias in the cancer group on the risk of stroke. Some studies showed that risk was consistent over time, and this may be related to the type of cancer; head and neck [23], Hodgkin lymphoma [25], and breast [45], or that risk declined and then increased again with breast cancer [12].

Increased stroke risk in cancer patients could also be a consequence of several cancer treatments. Studies have reported that platinum-based chemotherapy and angiogenesis inhibitors increase the risk of stroke [57–59]. This may occur because chemotherapy releases microparticles from cancer cells, which enhance thrombin generation [60]. In addition, radiotherapy can cause vasculopathy through accelerated atherosclerosis or other mechanisms, which can then precipitate stroke [61–63]. Several studies within this review looked at stroke incidence risk in relation to cancer treatment. Strongman’s analysis suggests cancer treatments, particularly chemotherapy, are likely to play a more prominent role than shared risk factors such as smoking or excess weight.

Patients with cervical cancer had decreased risk of stroke compared with the reference population [19]. It is suggested that supplementation of oestrogen after cancer treatment could explain this finding as hormone therapy has beneficial cardiovascular effects [64, 65]. A 45.5% of patients with cervical cancer took oestrogen supplementation compared with 15.5% in the control group. Several other studies, however, suggest increased cardiovascular morbidity following radiotherapy or chemoradiation in patients with cervical cancer [33, 43, 66]. Tsai et al. used the same database as Chang et al. to look at cervical cancer but in a group of patients who had radiotherapy treatment [43]. Patients who received radiation therapy as part of their cervical cancer treatment had a higher risk of ischaemic stroke compared with the general population. Radiotherapy in cervical cancer patients may induce systemic vascular damage that contributes to risk of stroke [67].

Hypertension and diabetes mellitus are predictors of ischemic stroke that remain significant into old age and have been shown to be risk factors in various cancer patients. Comorbidities in general may increase stroke risk much more for cancer patients than for controls. Cancer patients with diabetes, atrial fibrillation, or hypertension were at greater risk of stroke than those without [20, 29. 30, 40, 43]. In addition, cancer patients may be less likely to have primary and secondary stroke prevention.

### Strengths and limitations

Our review comprehensively demonstrates that adult cancer patients are at increased risk of stroke. It provides up-to-date results of the effect of cancer on the higher risk of stroke incidence by synthesising a number of published studies (*n* = 36). The study yielded a large population of individuals from countries in Asia, Europe, and North America.

We have focussed on adult cancers which we believe is a strength as the cancer types that develop in children differ in underlying pathology, behaviour, and treatment outcome compared to the much more commonly occurring tumours of middle and old age. Unlike many adult cancers, childhood cancers are not strongly linked to lifestyle or environmental risk factors, and long-term side effects are more of a concern. Different treatment protocols are used, and certain cancer treatments in childhood have moderate-to-severe late effects that require treatment and affect quality of life.

We chose to perform meta-analysis only on HR which account for both patient events and the time to events. Odds ratios or relative risks that measure only the number of events and take no account of when they occur are appropriate only for measuring dichotomous outcomes. Using such measures in a meta-analysis of time-to-event outcomes can pose problems and results in an estimate that is unreliable and difficult to interpret. Bias can also arise if the time points have been subjectively chosen.

The major limitation of this review is the high heterogeneity between the included studies. The review has brought together research conducted in different countries using different methodological approaches and with different follow-up times. All the studies identified are from the developed world. There were a substantial number of studies carried out in Taiwan (*n* = 11) all using the same research database, although these all investigated different cancer types. There was a range of different cancer types in the review with most studies focusing on survivors of a single, organ-specific cancer. There were eight studies looking at multiple cancer types.

The risk of stroke in those living with and beyond cancer is likely to be multifactorial and this review is limited in being able to distinguish between stroke caused by mechanisms related to cancer versus spontaneous non-cancer-related complications. Studies within this review generally used a retrospective matched cohort design using research databases or hospital databases to determine their patient cohort. They are therefore constrained and dependent on the quality of the collected data. Potential confounding variables that may account for some of the observations have not been included in analysis or may not have been collected within the research or hospital databases which may have led to biased estimates. Cancer and stroke share some common pathophysiological pathways and share several risk factors, including age, smoking, and obesity. Most studies within this review matched on age, sex, and specific individual comorbidities; however, many studies have stated that the limitations of their work are the absence of behavioural factors and lifestyle variables. Only five of the 30 studies adjusted for smoking. Although one study within the review showed a higher risk of ischemic and haemorrhagic stroke for cancers strongly associated with smoking [18], another study found that several non-smoking-related cancers were also associated with an increased risk of both stroke sub-types [6]. This risk declined rapidly after 6 months but remained raised for 10 or more years [6]. Several other studies within the review looked at smoking and increased stroke risk only within the cohort of cancer patients and found no association [25, 28, 38]. Smoking as a risk factor is unlikely to fully explain the association between cancer and stroke.

This review cannot account for effects of different socioeconomic status, racial differences, and country-specific treatment differences. Most studies did not include details on cancer stage, cancer treatment, or adjust for this in the analysis. There was also a lack of comprehensive clinical information on surgical intervention, dosage and location of radiotherapy, treatment duration and regimen of chemotherapy, and laboratory data in most studies within the review. Further research should explore these factors in detail.

### Implications and further research

This review presents strong evidence that patients with cancer are at increased risk of stroke. Stroke risk is particularly increased in individuals with leukaemia, myeloma, lung cancer, and pancreatic cancer. In addition to cancer type, cancer treatment is an important modifier of stroke risk; for example, those who have received platinum-based chemotherapy or who have received radiotherapy for head and neck cancer are at significantly increased risk of stroke. Further research is needed to explore mechanisms and provide specific guidance on how to minimise stroke risk in the growing population of cancer survivors. This includes studies on routinely collected data to explore behavioural and lifestyle factors, cancer stage, and treatment, prospective studies to identify biomarkers that can reliably predict first and recurrent stroke in cancer patients, translational studies to elucidate the mechanisms of these strokes, and clinical trials to identify the best strategies to prevent and acutely treat cerebrovascular events in the cancer population.

There is not yet sufficient evidence to provide a comprehensive patient-centred risk assessment tool for stroke that could be used in clinical settings with those living with and beyond cancer. Nonetheless, ensuring awareness of the link is important for both patients and clinicians, so that risk factors can be identified and modified. This review highlights the potential to use routinely collected healthcare data to develop and test new stroke risk calculators for individuals living with and beyond cancer. While there is not yet sufficient evidence to provide a comprehensive patient-centred risk assessment tool to use in clinical settings, clinicians should be aware that cancer can increase stroke risk and should use cancer care reviews as an opportunity to routinely discuss and address these modifiable risk factors for cardiovascular disease.

Primary prevention of cardiovascular diseases through the targeted management of modifiable risk factors such as hypertension, hypercholesterolaemia, diabetes, and lifestyle modification has been one of the major public health successes of the late twentieth century. However, current risk stratification tools such as QRISK and Framingham do not consider cancer or cancer treatment. More aggressive management of risk factors such as hypertension, obesity, and diabetes in cancer patients is needed. Patients with newly diagnosed malignancy should be routinely assessed and considered for antithrombotic and statin medicines for the primary prevention of cardiovascular disease. Given that patients with cancer are also prone to bleeding due to frequent coagulopathy and invasive procedures, carefully designed clinical trials are also needed to answer these questions.

This review highlights the need to use routinely collected healthcare data to develop and test new stroke risk calculators for individuals living with and beyond cancer and the need for clinical trials of primary prevention in individuals identified to be at high risk. Even with the limitations reported here, the development of comprehensive, evidence-based, national-level guidance on stroke in cancer survival care should be prioritised to optimise the care of this patient group.

## Conclusions

In conclusion, there is evidence that stroke incidence is significantly increased in those living with and beyond certain cancers. There is substantial heterogeneity between studies, and further research is needed to explore the mechanisms. Cardiovascular risk should be assessed during cancer survivorship care, with attention to modifying shared cancer/cardiovascular risk factors.

## Supplementary Information

Below is the link to the electronic supplementary material.

Supplementary file1 (DOCX 69 KB)

Supplementary file2 (DOC 66 KB)

## Data Availability

The data used and analysed during this study are available from the corresponding author.
