# Supplementary material for: Is stroke incidence increased in survivors of adult cancers? A systematic review and meta-analysis
Source: J Cancer Surviv. 2021 Nov 5;16(6):1414–48. doi: 10.1007/s11764-021-01122-7 (PMC9630245; doi:10.1007/s11764-021-01122-7)
Supplement: Supplementary file 1 — Supplementary file1 (DOCX 69 KB) [file 11764_2021_1122_MOESM1_ESM.docx]

**Is stroke incidence increased in survivors of adult cancers? A systematic review and meta-analysis**

**Melanie E. Turner^1^, Peter Murchie^1^, Sarah Derby^2^, Ariel Yuhan Ong^3^, Lauren Walji^4^, David McLernon^1^, Mary-Joan Macleod^5^, Rosalind Adam^1^**

^1^Institute of Applied Health Sciences, University of Aberdeen, Polwarth Building, Foresterhill, Aberdeen, AB25 2ZD, UK;

^2^Institute of Cancer Sciences, University of Glasgow, Wolfson Wohl Cancer Research Centre, Bearsden, Glasgow, G61 1BD, UK;

^3^Oxford Eye Hospital, Level Lg1 John Radcliffe Hospital, Headley Way, Headington, Oxford, OX3 9DU, UK;

^4^University of Aberdeen Medical School, Polwarth Building, Foresterhill, Aberdeen AB25 2ZD, UK;

^5^Institute of Medical Sciences, University of Aberdeen, Foresterhill, Aberdeen, AB25 2ZD, UK.

Corresponding Author: Dr Melanie Turner ([m.e.turner@abdn.ac.uk](mailto:m.e.turner@abdn.ac.uk)) +441224437136

Supplementary Figure 1. Search Strategy

Database: Ovid MEDLINE <2016 to April Week 3 2020> Search Strategy:

1 neoplasm/ (55230)

2 cancer$.tw. (301004)

3 1 or 2 (312802)

4 neurological.mp. (40154)

5 cerebrovascular.mp. (12679)

6 isch?emic.mp. (33782)

7 Cerebral Hemorrhage/ or Cerebrovascular Disorders/ or Stroke/ or Intracranial Hemorrhages/ (31877)

8 stroke/ (26635)

9 stroke.tw. (43324)

10 4 or 5 or 6 or 7 (95761)

11 8 or 9 (46203)

12 10 or 11 (108233)

13 ?etiology.mp. (279161)

14 incidence/ (47933)

15 outcome.mp. (366681)

16 risk factor*.mp. (205408)

17 confound*.mp. (27432)

18 13 or 14 or 15 or 16 or 17 (724548)

19 3 and 12 and 18 (1781)

20 remove duplicates from 19 (1780)

21 remove duplicates from 19 (1780)

22 20 not (comment or conference or letter).pt. (1774)

23 limit 21 to (english language and humans) (1634)

Supplementary Table 1. Study definitions of stroke

| **Study (author, year, reference)** | **Definition of stroke** |
| --- | --- |
| Adelborg et al, 2019, [16] | ICD-10 I63, I64 |
| Armenian et al, 2016, [17] | ICD-9 430-435, 00.61-00.65, 38.1, 39.74; ICD-10 I60-79 |
| Andersen et al, 2018, [18] | Defined according to the criteria of the WHO. |
| Chang et al, 2013, [19] | ICD-9 430-438 |
| Chan et al, 2018, [20] | Hemorrhagic stroke ICD-9 430-432; Ischemic stroke ICD-9 433-438 |
| Chen et al, 2011, [21] | ICD-9-CM codes 430-438, excluding traumatic strokes |
| Chia et al, 2013, [22] | ICD-9 code 430-437.1, 437.3-438.xx, |
| Chu et al, 2011, [23] | ICD-9-CM 433-438 or A-code A292-294 and A299 |
| Chu et al, 2013, [24] | ICD-9-CM 433-438 or A-code A292-294 and A299 |
| De Bruin et al, 2009, [25] | Verified by treating neurologists or general practitioners. |
| Dorresteijn et al, 2001, [26] | Fixed neurologic deficit lasting for more than 24 hours. Clinical diagnosis based on a neurologic examination, and in most cases, it was confirmed by a computed tomography or magnetic resonance imaging scan. |
| Haynes et al, 2002, [27] | Stroke defined as any definite acute cerebrovascular event resulting in permanent neurological deficit or the presence of nonspecific but persistent and significant neurological symptoms such as cognitive impairment with a confirmatory infarct(s) seen on head computed tomography (CT) or magnetic resonance imaging (MRI) scan. |
| Hooning et al, 2006, [28] | No detail on definition. Just states that data taken from medical record and GP or from oncologic records only. |
| Kuan et al, 2014, [29] | ICD-9-CM 436,433.X, 434.X, and 437.1X accompanied by computed tomographic or magnetic resonance images. |
| Kuan et al, 2015, [30] | ICD-9-CM code 436, 433.X, 434.X and 437.1X accompanied by computed tomographic or magnetic resonance images. |
| Lauritsen et al, 2019 [31] | ICD-10: I60-I64 (excl. I62) or ICD-8: 430-436 (excl.435) |
| Lee et al, 2011, [32] | ICD-9-CM codes 433-438 |
| Maduro et al, 2010, [33] | No detail on definition. Cerebrovascular accident. |
| Melloni et al, 2017, [34] | Defined as abrupt onset of a nontraumatic, focal neurologic deficit lasting at least 24 hours, or systemic embolism defined as symptoms consistent with acute loss of blood to a noncerebral artery confirmed by autopsy, angiography, vascular imaging, or other objective testing. |
| Moser et al, 2006, [35] | Cerebrovascular accident based on the International Classification of Health Problems in Primary Care (ICHPPC-2) |
| Moutsten et al, 2019, [36] | ICD-8 codes 43309, 43399, 43409, 43499, 43601, 43690; ICD-10 codes I63, I64 |
| Navi et al, 2015, [4] | ICD-9-CM codes ischemic stroke=433.x1, 434.x1 or 436; hemorrhagic stroke=430 or 431. |
| Nilsson et al, 2015, [12] | ICD-8 codes 430-438, 344; ICD-9 codes 430-438, 344,; ICD-10 codes I60-I67, I69, G45, G46 |
| Robinson et al,2012, [37] | ICD10 I60-I64, G45 |
| Shin et al, 2018, [38] | Defined as inpatient hospitalisation with ischemic stroke diagnosis |
| Shin et al, 2020, [39] | ICD-10 codes I63 or I64 during hospitalisation with claims for brain magnetic resonance imaging or brain computerized tomography. |
| Soisson et al, 2018, [40] | Provided in supplementary information, however unable to access this. |
| Strongman et al, 2019, [41] | ICD-10 codes I60, I61, I62, I63, I64, I67, I68, I69 |
| Suh et al, 2019, [42] | ICD-10 code I63 |
| Tsai et al, 2013, [43] | ICD-9-CM codes 433-438 |
| van-Hemelrijck et al, 2010, [44] | ICD-10-codes I60-I64, G45 |
| van Herk-Sukel et al, 2011, [45] | ICD-9-CM 433, 434, 437.1 |
| van Herk-Sukel et al, 2013, [46] | ICD-9-CM 433, 434, 437.1 |
| Wei et al, 2019, [47] | ICD9-CM codes 430, 431, 433, 434, 435. |
| Wu et al, 2014, [48] | ICD-9 CM codes 433-438 |
| Zoller et al, 2012, [6] | Ischaemic stroke ICD-9 433, 434, 435, 437.0, 437.1. ICD-10 I63 (not I636), I65, I66, I67.2 and I67.8; Haemorrhagic stroke ICD-9 431, 432. ICD-10 I61, I62 |

Supplementary Table 2. Incidence of stroke in relation to cancer treatment.

| **Reference group** | **Cancer Type** | **Study Ref** | **Cancer Treatment** | **Incidence of stroke** | **HR (95% CI)** | **SIR (95% CI)** |
| --- | --- | --- | --- | --- | --- | --- |
| Matched general population cohort | *Haematological* | Adelborg et al [16] | No CT | **↑** | 1.28 (1.15-1.43) |  |
|  |  |  | CT | ↔ | 1.14 (0.99-1.30) |  |
|  |  |  | No RT | **↑** | 1.26 (1.15-1.37) |  |
|  |  |  | RT | ↔ | 0.88 (0.64-1.21) |  |
|  | *Non-Hodgkin lymphoma* | Strongman et al [41] | No CT/RT | **↑** | 1.61 (1.18-2.21) |  |
|  |  |  | CT | **↑** | 1.11 (1.01-1.95) |  |
|  |  |  | RT | ↔ | 1.46 (0.75-2.84) |  |
|  |  |  | Both CT/RT | ↔ | 1.38 (0.81-2.36) |  |
|  |  | Moser et al [35] | Salvage treatment | ↔ |  | 1.4 (0.5-2.8) |
|  |  |  | No salvage treatment | ↔ |  | 1.3 (0.3-3.2) |
|  |  |  | RT | ↔ |  | 2.3 (1.0-4.5) |
|  |  |  | No RT | ↔ |  | 0.6 (0.1-1.5) |
|  |  |  | ASCT | 0 |  | 0 |
|  |  |  | No ASCT | ↔ |  | 1.4 (0.7-2.5) |
|  | *Hodgkin lymphoma* | De Bruin et al [25] | RT | **↑** |  | 2.0 (1.2-3.1) |
|  |  |  | CT | ↔ |  | 0.4 (0.0-2.1) |
|  |  |  | RT/CT | **↑** |  | 2.6 (1.9-3.5) |
|  | *Nasopharyngeal* | Chu et al [24] | RT | **↑** | 1.90 (1.53-2.35) |  |
|  |  |  | RT/CT | **↑** | 2.59 (2.21-3.03) |  |
|  |  |  | Non-RT/CT | **↑** | 1.70 (1.35-2.15) |  |
|  | *Ovarian* | Kuan et al [29] | Any CT | **↑** | 1.45 (1.07-1.97) |  |
|  |  |  | Cisplatin-based CT | **↑** | 1.38 (1.07-1.76) |  |
|  |  |  | Carboplatin-based CT | **↑** | 1.46 (1.13-1.89) |  |
|  |  |  | Non-platinum-based CT | ↔ | 1.12 (0.61-2.04) |  |
|  | *Gastric* | Kuan et al [30] | Major surgery | **↑** | 1.56 (1.36-1.79) |  |
|  |  |  | CT | ↔ | 1.06 (0.93-1.21) |  |
|  |  |  | RT | ↔ | 0.90 (0.73-1.10) |  |
|  | *Colorectal* | Strongman et al [41] | No CT/RT | ↔ | 1.00 (0.94-1.20) |  |
|  |  |  | CT | ↔ | 0.91 (0.72-1.16) |  |
|  |  |  | RT | ↔ | 1.18 (0.76-1.81) |  |
|  |  |  | Both CT/RT | ↔ | 1.14 (0.80-1.64) |  |
|  | *Prostate* | Moutsten et al [36] | Active surveillance | ↔ | 0.25 (0.03-1.76) |  |
|  |  |  | Watchful waiting | ↔ | 0.63 (0.30-1.33) |  |
|  |  |  | Curative intended treatment | ↔ | 0.75 (0.51-1.12) |  |
|  |  |  | Palliative treatment | **↑** | 2.09 (1.49-2.93) |  |
|  |  | Robinson et al [37] | No endocrine treatment | ↔ | 1.10 (1.00-1.21) |  |
|  |  |  | Any endocrine treatment | ↔ | 1.16 (0.97-1.38) |  |
|  |  |  | GnRH agonists | **↑** | 1.39 (1.08-1.77) |  |
|  |  |  | GnRH & AA | ↔ | 0.78 (0.46-1.32) |  |
|  |  |  | AA | ↔ | 1.19 (0.79-1.78) |  |
|  |  |  | Other endocrine treatment | ↔ | 0.76 (0.48-1.21) |  |
|  |  | Shin et al [39] | Active surveillance/watchful waiting | ↔ | 0.93 (0.84-1.04) |  |
|  |  |  | Surgery | ↓ | 0.73 (0.66-0.80) |  |
|  |  |  | Surgery & ADT | ↓ | 0.78 (0.67-0.91) |  |
|  |  |  | RT & ADT | ↔ | 0.83 (0.58-1.18) |  |
|  |  |  | ADT only | ↔ | 1.03 (0.97-1.10) |  |
|  |  |  | RT only | ↔ | 0.91 (0.52-1.61) |  |
|  |  | van-Hemelrijck et al [44] | Surveillance | **↑** |  | 1.19 (1.14-1.25) |
|  |  |  | Curative treatment | ↔ |  | 0.97 (0.90-1.04) |
|  |  |  | Any Endocrine treatment | **↑** |  | 1.24 (1.18-1.30) |
|  |  |  | Anti-androgens | **↑** | 1.19 (1.02-1.40) |  |
|  |  |  | Orchiectomy | **↑** | 1.19 (1.02-1.37) |  |
|  |  |  | GnRH Agonists | **↑** | 1.27 (1.12-1.43) |  |
|  |  |  | GnRH & AA | **↑** | 1.36 (1.20-1.55) |  |
|  |  | Strongman et al [41] | No CT/RT | ↔ | 1.08 (0.98-1.18) |  |
|  |  |  | CT | ↔ | 0.88 (0.46-1.68) |  |
|  |  |  | RT | ↔ | 1.02 (0.88-1.19) |  |
|  |  |  | Both CT/RT | ↔ | 0.85 (0.50-1.44) |  |
|  | *Lung* | Strongman et al [41] | No CT/RT | ↔ | 1.34 (1.00-1.79) |  |
|  |  |  | CT | **↑** | 1.87 (1.08-3.26) |  |
|  |  |  | RT | **↑** | 1.79 (1.17-2.75) |  |
|  |  |  | Both CT/RT | **↑** | 1.96 (1.23-3.14) |  |
|  |  | van Herk-Sukel et al [46] | Surgery | ↔ | 1.20 (0.40-4.00) |  |
|  |  |  | CT | ↔ | 1.60 (0.40-6.60) |  |
|  | *Breast* | Strongman et al [41] | No CT/RT | ↔ | 1.12 (0.99-1.27) |  |
|  |  |  | CT | ↔ | 0.75 (0.49-1.14) |  |
|  |  |  | RT | ↔ | 0.97 (0.83-1.12) |  |
|  |  |  | Both CT/RT | ↔ | 1.21 (0.91-1.60) |  |
|  |  | Hooning et al [28] | Surgery only | ↓ |  | 0.61 (0.39-0.91) |
|  |  |  | RT ± surgery | ↓ |  | 0.67 (0.54-0.82) |
|  |  |  | RT + CT ± surgery | ↓ |  | 0.94 (0.45-1.72) |
|  |  |  | RT + HT ± surgery | ↔ |  | 1.31 (0.87-1.88) |
|  |  |  | RT + CT + HT ± surgery | ↓ |  | 0.83 (0.34-1.72) |
|  | *Uterus* | Strongman et al [41] | No CT/RT | ↔ | 1.15 (0.87-1.51) |  |
|  |  |  | RT | ↔ | 1.06 (0.71-1.58) |  |
|  |  |  | CT ± RT | ↔ | 1.24 (0.49-3.16) |  |
|  | *Cervical* | Maduro et al [33] | RT & CT | ↔ |  | 0.70 (0.14-2.05) |
|  |  |  | RT | ↔ |  | 0.63 (0.20-1.47) |
|  | *Thyroid* | Suh et al [42] | Unilateral lobectomy | ↔ | 1.05 (0.80-1.38) |  |
|  |  |  | Total thyroidectomy | **↑** | 1.16 (1.10-1.22) |  |
|  |  |  | Radioiodine ablation (-) | **↑** | 1.18 (1.09-1.28) |  |
|  |  |  | Radioiodine ablation (+) | **↑** | 1.13 (1.06-1.21) |  |
|  | *Testicular* | Lauritsen et al [31] | Surveillance  <1yr  1-10yrs  >10yrs | ↔  ↔  ↔ | 0.8 (0.3-1.8)  0.9 (0.6-1.3)  0.8 (0.6-1.2) |  |
|  |  |  | BEP  <1yr  1-10yrs  >10yrs | ↑  ↔  ↔ | 6.0 (2.6-14.1)  1.1 (0.7-1.9)  1.2 (0.8-1.7) |  |
|  |  |  | RT  <1yr  1-10yrs  >10yrs | -  ↔  ↔ | -  0.7 (0.4-1.6)  0.9 (0.5-1.5) |  |
| Cancer cohort who had surgery | *Cervical* | Chang et al [19] | RT | **↑** | 1.88 (1.52-2.32) |  |
|  |  |  | CT | ↔ | 0.89 (0.71-1.11) |  |
|  |  |  | RT & CT | ↔ | 0.94 (0.78-1.15) |  |
| Cancer cohort who had no RT or CT | *Head and neck* | Chu et al [23] | RT | ↔ | 1.10 (0.89-1.35) |  |
|  |  |  | CT | ↔ | 1.31 (1.00-1.35) |  |
|  |  |  | RT/CT | ↑ | 1.46 (1.22-1.74) |  |
| Cancer cohort who had no CT | *Gastric* | Shin et al [38] | CT | ↔ | 0.89 (0.74-1.06) |  |
| Cancer cohort active surveillance/watchful waiting | *Prostate* | Shin et al [39] | Surgery | ↓ | 0.75 (0.65-0.87) |  |
|  |  |  | Surgery & ADT | ↓ | 0.81 (0.68-0.98) |  |
|  |  |  | RT & ADT | ↔ | 0.88 (0.61-1.28) |  |
|  |  |  | ADT only | ↑ | 1.16 (1.02-1.32) |  |
|  |  |  | RT only | ↔ | 0.98 (0.55-1.74) |  |
| Cancer cohort who had surgery alone | *Oral* | Wu et al [48] | RT/CT/CCRT | ↑ | 1.24 (1.10-1.40) |  |
|  |  |  | Surgery & adjuvant therapy | ↔ | 1.08 (0.92-1.27) |  |

HR=Hazard Ratio; CI=Confidence Interval; RR=Relative Risk; SIR=Standardised incidence ratio; CT=Chemotherapy; RT=Radiotherapy; ASCT=autologous stem cell transplantation; GnRH = gonadotrophin-releasing hormone; AA=antiandrogens; ADT=androgen deprivation therapy; HT=Hormone therapy; BEP= bleomycin-etoposide-cisplatin; CCRT= concurrent chemoradiotherapy; ↑=increase; ↔ = no difference; ↓=decrease.

Supplementary Table 3. Incidence of stroke in relation to time since cancer diagnosis.

| **Cancer Type** | **Time period** | **Study (author, reference)** | **Incidence of stroke** | **HR (95% CI)** | **RR (95% CI)** | **SIR (95% CI)** |
| --- | --- | --- | --- | --- | --- | --- |
| ***Hodgkin lymphoma*** | 0-6mths | Adelborg et al [16] | ↓ | 0.26 (0.07-0.72) |  |  |
|  |  | Zoller et al [6] | ↔ |  |  | 1.3 (0.4-3.0) |
|  | >6-12mths | Zoller et al [6] | ↔ |  |  | 2.2 (0.9-4.6) |
|  | >1yr-5yrs | Adelborg et al [16] | ↔ | 1.12 (0.52-2.15) |  |  |
|  |  | Zoller et al [6] | ↔ |  |  | 1.3 (0.8-1.9) |
|  | >5-10yrs | Adelborg et al [16] | ↔ | 0.57 (0.12-1.91) |  |  |
|  |  | Zoller et al [6] | ↔ |  |  | 0.8 (0.4-1.4) |
|  |  | De Bruin et al [25] | ↑ |  |  | 2.1 (1.0-3.8) |
|  | >10yrs | Zoller et al [6] | ↔ |  |  | 1.5 (0.9-2.4) |
|  | 10-14yrs | De Bruin et al [25] | ↑ |  |  | 2.3 (1.3-3.9) |
|  | 15-19yrs |  | ↑ |  |  | 2.6 (1.5-4.3) |
|  | 20-24yrs |  | ↑ |  |  | 2.1 (1.0-3.7) |
|  | >25yrs |  | ↑ |  |  | 1.9 (1.0-3.7) |
| ***Non-Hodgkin lymphoma*** | 0-6mths | Adelborg et al [16] | ↓ | 0.41 (0.30-0.56) |  |  |
|  |  | Zoller et al [6] | ↑ |  |  | 1.6 (1.4-1.8) |
|  | >6-12mths | Adelborg et al [16] | ↓ | 0.23 (0.14-0.36) |  |  |
|  |  | Zoller et al [6] | ↔ |  |  | 1.1 (0.9-1.3) |
|  | 1-2yrs | Strongman et al [41] | ↔ | 1.37 (0.92-2.06) |  |  |
|  | >1-5yrs | Adelborg et al [16] | ↑ | 1.57 (1.25-1.94) |  |  |
|  |  | Zoller et al [6] | ↔ |  |  | 1.0 (0.9-1.1) |
|  | 2-5yrs | Strongman et al [41] | ↑ | 1.54 (1.18-2.01) |  |  |
|  | >5-10yrs | Adelborg et al [16] | ↑ | 3.78 (2.14-6.14) |  |  |
|  |  | Zoller et al [6] | ↔ |  |  | 1.2 (1.0-1.3) |
|  | 5yrs+ | Strongman et al [41] | ↑ | 1.46 (1.11-1.94) |  |  |
|  | >10yrs | Zoller et al [6] | ↑ |  |  | 1.2 (1.1-1.4) |
| ***Acute myeloid leukaemia*** | 0-6mths | Adelborg et al [16] | ↓ | 0.32 (0.16-0.59) |  |  |
|  | >6-12mths |  | ↓ | 0.21 (0.06-0.58) |  |  |
|  | >1yr -5yrs |  | ↔ | 1.54 (0.88-2.52) |  |  |
|  | >5-10yrs |  | ↔ | 0.75 (0.15-2.51) |  |  |
| ***Acute lymphoid leukaemia*** | 0-6mths | Adelborg et al [16] | ↔ | 0.24 (0.02-1.29) |  |  |
| ***Chronic myeloid leukaemia*** | 0-6mths | Adelborg et al [16] | ↓ | 0.12 (0.01-0.63) |  |  |
|  | >6-12mths |  | ↓ | 0.29 (0.06-0.98) |  |  |
|  | >1yr -5yrs |  | ↔ | 0.57 (0.16-1.60) |  |  |
|  | >5-10yrs |  | ↔ | 0.53 (0.05-2.72) |  |  |
| ***Chronic lymphocytic leukaemia*** | 0-6mths | Adelborg et al [16] | ↓ | 0.48 (0.31-0.71) |  |  |
|  | >6-12mths |  | ↓ | 0.26 (0.14-0.52) |  |  |
|  | >1yr -5yrs |  | ↑ | 2.13 (1.68-2.66) |  |  |
|  | >5-10yrs |  | ↑ | 1.75 (1.10-2.64) |  |  |
| ***Leukaemia*** | <6mths | Zoller et al [6] | ↑ |  |  | 3.0 (2.5-3.7) |
|  | 6-12mths | Zoller et al [6] | ↔ |  |  | 1.2 (0.8-1.7) |
|  | 1-2yrs | Strongman et al [41] | ↑ | 1.81 (1.26-2.61) |  |  |
|  | 1-5yrs | Zoller et al [6] | ↔ |  |  | 1.1 (0.9-1.3) |
|  | 2-5yrs | Strongman et al [41] | ↑ | 1.40 (1.05-1.88) |  |  |
|  | >5yrs | Strongman et al [41] | ↔ | 1.09 (0.78-1.53) |  |  |
|  | 5-10yrs | Zoller et al [6] | ↑ |  |  | 1.5 (1.2-1.8) |
|  | >10yrs | Zoller et al [6] | ↔ |  |  | 1.3 (0.9-1.8) |
| ***Multiple myeloma*** | 0-6mths | Adelborg et al [16] | ↓ | 0.48 (0.30-0.73) |  |  |
|  | 0-6mths | Zoller et al [6] | ↑ |  |  | 1.5 (1.1-2.0) |
|  | >6-12mths | Adelborg et al [16] | ↓ | 0.28 (0.14-0.52) |  |  |
|  | >6-12mths | Zoller et al [6] | ↑ |  |  | 0.8 (0.5-1.2) |
|  | >1-5 yrs | Adelborg et al [16] | ↑ | 2.11 (1.59-2.75) |  |  |
|  | >1-5yrs | Zoller et al [6] | ↔ |  |  | 1.0 (0.9-1.2) |
|  | >5-10 yrs | Adelborg et al [16] | ↔ | 1.12 (0.53-2.13) |  |  |
|  | >5-10yrs | Zoller et al [6] | ↔ |  |  | 1.0 (0.8-1.4) |
|  | >10yrs | Zoller et al [6] | ↔ |  |  | 1.1 (0.6-1.8) |
| ***Head & Neck*** | <2yrs | Chu et al [23] | ↑ | 1.64 (1.42-1.90) |  |  |
|  | 2-4yrs |  | ↑ | 1.26 (1.05-1.50) |  |  |
|  | 5-6yrs |  | ↑ | 1.53 (1.29-1.81) |  |  |
|  | 7-8yrs |  | ↑ | 1.97 (1.61-2.41) |  |  |
|  | >8yrs |  | ↑ | 1.97 (1.03-3.78) |  |  |
|  | 0-9yrs | Dorresteijn et al [26] | ↑ |  | 3.7 (1.3-8.0) |  |
|  | >10yrs |  | ↑ |  | 10.1 (4.4-20.0) |  |
| ***Cervical cancer*** | <6mths | Zoller et al [6] | ↔ |  |  | 1.6 (1.0-2.4) |
|  | >6-12mths |  | ↔ |  |  | 1.3 (0.8-2.2) |
|  | <1yr | Chang et al [19] | ↓ | 0.34 (0.30-0.39) |  |  |
|  | 1-2yrs |  | ↓ | 0.64 (0.54-0.74) |  |  |
|  | 2-3yrs |  | ↓ | 0.52 (0.43-0.63) |  |  |
|  | >3yrs |  | ↓ | 0.77 (0.70-0.83) |  |  |
|  | >1-5yrs | Zoller et al [6] | ↔ |  |  | 1.2 (1.0-1.6) |
|  | >5-10yrs |  | ↔ |  |  | 1.3 (1.0-1.6) |
|  | >10yrs |  | ↔ |  |  | 1.1 (0.8-1.4) |
| ***Endometrial cancer*** | 1-5yrs | Soisson et al [41] | ↔ | 1.22 (0.80-1.87) |  |  |
|  | 5-10yrs |  | ↔ | 1.07 (0.66-1.74) |  |  |
| ***Lung*** | 0-1mths | Navi et al [4] | ↑ | 7.43 (6.65-2.89) |  |  |
|  | 1-3mths |  | ↑ | 2.66 (2.42-2.91) |  |  |
|  | 3-6mths |  | ↑ | 1.95 (1.79-2.12) |  |  |
|  | 0-6mths | van Herk-Sukel et al [46] | ↔ | 1.60 (0.70-4.00) |  |  |
|  | <6mths | Zoller et al [6] | ↑ |  |  | 2.2 (1.9-2.4) |
|  | 6-9mths | Navi et al [4] | ↑ | 1.63 (1.47-1.80) |  |  |
|  | 9-12mths | Navi et al [4] | ↑ | 1.69 (1.51-1.88) |  |  |
|  | >6mths | van Herk-Sukel et al [46] | ↔ | 0.70 (0.40-1.30) |  |  |
|  | 6-12mths | Zoller et al [6] | ↑ |  |  | 1.3 (1.0-1.6) |
|  | 0-1yr | Chen et al [21] | ↑ | Men 1.81 (1.63-2.01)  Women 2.35 (2.02-2.74) |  |  |
|  | 1-2yrs | Strongman et al [41] | ↑ | 2.06 (1.56-2.73) |  |  |
|  | 1-2yrs | Chen et al [21] | ↔ for men  ↑ for women | Men 1.06 (0.89-1.26)  Women 1.55 (1.23-1.94) |  |  |
|  | 2-3yrs |  | ↔ | Men 0.86 (0.66-1.12)  Women 0.88 (0.61-1.29) |  |  |
|  | >3yrs |  | ↔ | Men 1.04 (0.87-1.25)  Women 0.91 (0.69-1.20) |  |  |
|  | 1-5yrs | Zoller et al [6] | ↔ |  |  | 1.1 (0.99-1.3) |
|  | 2-5yrs | Strongman et al [41] | ↑ | 1.36 (1.01-1.84) |  |  |
|  | >5yrs | Strongman et al [41] | ↔ | 1.04 (0.71-1.53) |  |  |
|  | 5-10yrs | Zoller et al [6] | ↔ |  |  | 1.1 (0.9-1.3) |
|  | >10yrs |  | ↔ |  |  | 1.3 (1.0-1.6) |
| ***Breast*** | 0-1mths | Navi et al [4] | ↑ | 1.71 (1.48-1.99) |  |  |
|  | 1-3mths |  | ↑ | 1.17 (1.03-1.32) |  |  |
|  | 3-6mths |  | ↓ | 0.86 (0.77-0.96) |  |  |
|  | 0-6mths | van Herk-Sukel et al [45] | ↔ | 1.1 (0.5-2.5) |  |  |
|  | <6mths | Zoller et al [6] | ↑ |  |  | 1.5 (1.3-1.6) |
|  | 6-9mths | Navi et al [4] | ↔ | 0.92 (0.82-1.04) |  |  |
|  | 9-12mths |  | ↔ | 0.93 (0.83-1.04) |  |  |
|  | 6-12mths | van Herk-Sukel et al [45] | ↔ | 1.8 (0.8-3.9) |  |  |
|  | 6-12mths | Zoller et al [6] | ↑ |  |  | 1.2 (1.0-1.3) |
|  | <12mths | Nilsson et al [12] | ↑ |  | 1.22 (1.06-1.39) |  |
|  | >12mths | van Herk-Sukel et al [45] | ↔ | 1.2 (0.9-1.6) |  |  |
|  | 1-2yrs | Strongman et al [41] | ↔ | 1.13 (0.93-1.36) |  |  |
|  | 1-5yrs | Nilsson et al [12] | ↔ |  | 1.04 (0.96-1.13) |  |
|  | 1-5yrs | Zoller et al [6] | ↔ |  |  | 1.1 (1.0-1.1) |
|  | 2-5yrs | Strongman et al [41] | ↔ | 0.98 (0.86-1.12) |  |  |
|  | 5-10yrs | Nilsson et al [12] | ↑ |  | 1.17 (1.07-1.27) |  |
|  | 5-10yrs | Zoller et al [6] | ↑ |  |  | 1.1 (1.0-1.1) |
|  | >5yrs | Strongman et al [41] | ↑ | 1.13 (1.01-1.28) |  |  |
|  | >10yrs | Nilsson et al [12] | ↑ |  | 1.14 (1.04-1.25) |  |
|  | >10yrs | Zoller et al [6] | ↑ |  |  | 1.1 (1.0-1.1) |
|  | 10-14yrs | Hooning et al [28] | ↓ |  |  | 0.60 (0.45-0.79) |
|  | 15-19yrs |  | ↔ |  |  | 0.93 (0.72-1.17) |
|  | >20yrs |  | ↔ |  |  | 0.75 (0.54-1.01) |
| ***Colorectal*** | 0-1mths | Navi et al [4] | ↑ | 4.16 (3.66-4.72) |  |  |
|  | 1-3mths |  | ↑ | 1.80 (1.62-2.00) |  |  |
|  | 3-6mths |  | ↑ | 1.37 (1.25-1.51) |  |  |
|  | <6mths | Zoller et al [6] | ↑ |  |  | 1.6 (1.5-1.8) |
|  | 6-9mths | Navi et al [4] | ↔ | 0.92 (0.83-1.03) |  |  |
|  | 9-12mths |  | ↓ | 0.85 (0.75-0.95) |  |  |
|  | 6-12mths | Zoller et al [6] | ↔ |  |  | 1.1 (1.0-1.3) |
|  | 1-2yrs | Strongman et al [41] | ↔ | 1.15 (0.95-1.40) |  |  |
|  | 1-5yrs | Zoller et al [6] | ↑ |  |  | 1.2 (1.1-1.3) |
|  | 2-5yrs | Strongman et al [41] | ↔ | 1.06 (0.92-1.23) |  |  |
|  | >5yrs |  | ↔ | 1.02 (0.87-1.18) |  |  |
|  | 5-10yrs | Zoller et al [6] | ↑ |  |  | 1.2 (1.1-1.3) |
|  | >10yrs |  | ↑ |  |  | 1.3 (1.2-1.4) |
| ***Prostate*** | 0-1mths | Navi et al [4] | ↑ | 1.25 (1.09-1.43) |  |  |
|  | 1-3mths |  | ↔ | 0.97 (0.88-1.08) |  |  |
|  | 3-6mths |  | ↔ | 0.96 (0.88-1.05) |  |  |
|  | 0-6mths | Zoller et al [6] | ↑ |  |  | 1.2 (1.1-1.3) |
|  | 6-9mths | Navi et al [4] | ↓ | 0.90 (0.81-0.99) |  |  |
|  | 9-12mths |  | ↔ | 0.93 (0.84-1.03) |  |  |
|  | 6-12mths | Zoller et al [6] | ↑ |  |  | 1.1 (1.0-1.2) |
|  | 1-2yrs | Strongman et al [41] | ↔ | 1.06 (0.91-1.23) |  |  |
|  | 1-5yrs | Zoller et al [6] | ↑ |  |  | 1.2 (1.1-1.2) |
|  | 2-5yrs | Strongman et al [41] | ↔ | 1.07 (0.96-1.20) |  |  |
|  | >5yrs |  | ↔ | 1.05 (0.93-1.18) |  |  |
|  | 5-10yrs | Zoller et al [6] | ↑ |  |  | 1.2 (1.2-1.3) |
|  | >10yrs |  | ↑ |  |  | 1.2 (1.1-1.3) |
| ***Pancreas*** | 0-1mths | Navi et al [4] | ↑ | 4.25 (3.32-5.45) |  |  |
|  | 1-3mths |  | ↑ | 2.14 (1.73-2.65) |  |  |
|  | 3-6mths |  | ↑ | 1.62 (1.31-2.01) |  |  |
|  | 0-6mths | Zoller et al [6] | ↑ |  |  | 2.2 (1.8-2.7) |
|  |  | Chan et al [20] | ↑ | 4.37 (3.45-5.54) |  |  |
|  | 7-12 mths |  | ↑ | 1.87 (1.21-2.90) |  |  |
|  | 1-2yrs |  | ↑ | 2.01 (1.33-3.06) |  |  |
|  | >2yrs |  | ↔ | 0.79 (0.35-1.77) |  |  |

HR=hazard ratio; CI=confidence intervals; RR=relative risk; SIR=standardised incidence ratio; mths=months; yrs=years; ↑=increase; ↔ = no difference; ↓=decrease.

Supplementary Table 4. Incidence of stroke in relation to smoking status.

| **Reference group** | **Cancer Type** | **Study (author, reference)** | **Smoking status** | **Incidence of stroke** | **HR (95% CI)** | **RR (95% CI)** | **SIR (95% CI)** |
| --- | --- | --- | --- | --- | --- | --- | --- |
| Matched general population cohort | *Smoking related (bladder; colon; head & neck; kidney; lung; pancreas; rectum; stomach)* | Andersen et al [18] |  | **↑** | Ischaemic 1.67 (1.51-1.83)  Haemorrhagic 1.50 (1.08-2.07**)** |  |  |
|  | *Non-smoking related (breast; endometrial; melanoma; non-Hodgkin; ovarian; prostate)* | Andersen et al [18] |  | ↔ | Ischaemic 1.10 (0.98-1.24)  Haemorrhagic 1.23 (0.87-1.76) |  |  |
|  | *Head & neck* | Dorresteijn et al [26] | Smoker | **↑** |  | 5.7 (2.1-12.5) |  |
|  |  |  | Non-Smoker | **↑** |  | 6.4 (2.7-12.5) |  |
|  | *non-Hodgkin lymphoma* | Moser et al [35] | Smoker | ↔ |  |  | 1.9 (0.6-4.3) |
|  |  |  | Non-smoker | ↔ |  |  | 1.5 (0.6-3.3) |
|  |  | Strongman et al [41] | Never smoked | **↑** | 1.68 (1.30-2.17) |  |  |
|  |  |  | Ever smoked | **↑** | 1.31 (1.01-1.69) |  |  |
|  | *Colorectal* | Strongman et al [41] | Never smoked | ↔ | 0.99 (0.86-1.14) |  |  |
|  |  |  | Ever smoked | ↔ | 1.13 (0.99-1.29) |  |  |
|  | *Lung* | Strongman et al [41] | Never smoked | ↔ | 1.44 (0.89-2.35) |  |  |
|  |  |  | Ever smoked | **↑** | 1.53 (1.24-1.87) |  |  |
|  | *Malignant melanoma* | Strongman et al [41] | Never smoked | **↑** | 1.30 (1.05-1.61) |  |  |
|  |  |  | Ever smoked | ↔ | 0.90 (0.70-1.14) |  |  |
|  | *Breast* | Strongman et al [41] | Never smoked | ↔ | 0.96 (0.86-1.07) |  |  |
|  |  |  | Ever smoked | **↑** | 1.24 (1.09-1.40) |  |  |
|  | *Uterus* | Strongman et al [41] | Never smoked | ↔ | 1.27 (0.97-1.67) |  |  |
|  |  |  | Ever smoked | ↔ | 0.92 (0.63-1.36) |  |  |
|  | *Prostate* | Strongman et al [41] | Never smoked | ↔ | 1.01 (0.90-1.14) |  |  |
|  |  |  | Ever smoked | ↔ | 1.09 (1.00-1.20) |  |  |
|  | *Bladder* | Strongman et al [41] | Never smoked | ↔ | 1.01 (0.82-1.24) |  |  |
|  |  |  | Ever smoked | ↔ | 1.10 (0.95-1.26) |  |  |
|  | *Leukaemia* | Strongman et al [41] | Never smoked | ↔ | 1.16 (0.87-1.56) |  |  |
|  |  |  | Ever smoked | **↑** | 1.61 (1.23-2.11) |  |  |
| Cancer cohort who had never smoked | *Hodgkin lymphoma* | De Bruin et al [25] | Past smoker | ↔ | 0.9 (0.5-1.4) |  |  |
|  |  |  | Current smoker | ↔ | 1.2 (0.7-2.3) |  |  |
|  | *Breast* | Hooning et al [28] | Past smoker | ↔ | 1.00 (0.58-1.72) |  |  |
|  |  |  | Current smoker | ↔ | 1.37 (0.96-1.95) |  |  |
|  | *Gastric* | Shin et al [38] | Past smoker | ↔ | 0.99 (0.82-1.20) |  |  |
|  |  |  | Current smoker | ↔ | 1.10 (0.94-1.30) |  |  |
|  | *Thyroid* | Suh et al [42] | Past smoker | ↔ | 1.10 (0.87-1.40) |  |  |
|  |  |  | Current smoker | **↑** | 1.28 (1.04-1.58) |  |  |

HR=Hazard Ratio; CI=Confidence Interval; RR=Relative Risk; SIR=Standardised incidence ratio; ↑=increase; ↔ = no difference; ↓=decrease.

Supplementary Table 5. Incidence of stroke in studies using Incidence Rate Ratio

| **Study (author, year, reference)** | **Control group** | **Cancer group** | **Control group incidence rate** | **Cancer group incidence rate** | **Crude IRR (CI)**  **(p-value)** | **Adjusted IRR (CI) (p-value)** |
| --- | --- | --- | --- | --- | --- | --- |
| **Armenian et al, 2016, [17]** |  |  |  |  |  |  |
| *Overall* | 3493/73,545 | 1704/36,232^1^ | 10.87 (per 1000 person years) | 11.12 (per 1000 person years) | 1.02 (0.97-1.09)  p=0.44 | 0.98 (0.93-1.04)  p=0.54 |
| *Bladder cancer* | 85 | 38 | 18.06 | 19.63 | 1.09 (0.74-1.59) p=0.67 | 0.93 (0.62-1.39) p=0.73 |
| *Breast cancer* | 838 | 454 | 8.66 | 9.71 | 1.12 (1.00-1.26) p=0.05 | 1.07 (0.95-1.20) p=0.25 |
| *Chronic Lymphocytic Leukaemia* | 73 | 22 | 16.75 | 11.18 | 0.67 (0.41-1.08) p=0.10 | 0.62 (0.38-1.01) p=0.06 |
| *Colon* | 299 | 117 | 12.74 | 11.65 | 0.91 (0.74-1.13) p=0.41 | 0.90 (0.73-1.12) p=0.36 |
| *Kidney* | 96 | 56 | 9.69 | 13.73 | 1.42 (1.02-1.97) p=0.04 | 1.38 (0.99-1.94) p=0.06 |
| *Lung & Bronchus* | 172 | 82 | 14.51 | 26.32 | 1.81 (1.39-2.36) p<0.01 | 1.85 (1.36-2.52) p<0.01 |
| *Melanoma* | 185 | 96 | 9.57 | 10.25 | 1.07 (0.84-1.37) p=0.58 | 1.09 (0.85-1.41) p=0.49 |
| *Multiple myeloma* | 47 | 26 | 11.91 | 21.67 | 1.82 (1.13-2.94) p=0.01 | 1.83 (1.11-3.00) p=0.02 |
| *Non-Hodgkin Lymphoma* | 184 | 75 | 12.7 | 12.42 | 0.98 (0.75-1.28) p=0.87 | 1.03 (0.78-1.35) p=0.83 |
| *Ovary* | 39 | 27 | 5.13 | 8.69 | 1.69 (1.04-2.76) p=0.04 | 1.64 (0.99-2.71) p=0.05 |
| *Prostate* | 1178 | 584 | 13.4 | 12.27 | 0.92 (0.83-1.01) p=0.08 | 0.87 (0.78-0.96) p<0.01 |
| *Rectum/recto-sigmoid* | 96 | 43 | 8.76 | 8.38 | 0.96 (0.67-1.37) p=0.81 | 0.85 (0.59-1.24) p=0.41 |
| *Thyroid* | 56 | 26 | 5.74 | 5.1 | 0.89 (0.56-1.42) p=0.62 | 0.85 (0.53-1.38) p=0.52 |
| *Uterus* | 145 | 58 | 8.87 | 7.39 | 0.83 (0.61-1.13) p=0.24 | 0.81 (0.59-1.10) p=0.18 |
| **Chia et al, 2013, [22]**  *(Ovarian cancer)* |  |  |  |  |  |  |
| *3-month incidence* |  |  | 32.3 per 1000 person years  (95% CI 20.9-43.8) | 74.0 per1000 person years  (95% CI 57.8-90.1) |  |  |
| *12-month incidence* |  |  | 23.4 per 1000 person years  (95% CI 18.8-28.0) | 35.8 per 1000 person years  (95% CI 29.5-42.0) |  |  |
|  |  |  |  |  |  | **RR (95% CI)** |
| **Dorresteijn et al, 2001, [26]**  *(Head & Neck cancer)* |  | 14/367 |  |  |  | 5.6 (3.1-9.4) |
|  |  |  |  | **5-year actuarial rate of stroke** |  | **RR (95% CI) (p-value)** |
| **Haynes et al, 2002, [27]**  *(Head & Neck cancer)* |  | 20/413 |  | 12% |  | 2.09 (1.28-3.22) (p=0.0007) |

IRR=Incidence Rate Ratio; CI=Confidence Interval

Supplementary Table 6. Incidence of stroke in studies using standardised incidence ratio, absolute excess risk, and relative risk.

| **Study (author, year)** | **Control group** | **Cancer group** | **Standardized incidence ratio (95% CI)** | **Absolute excess risk (95% CI)** | **RR (95% CI)** |
| --- | --- | --- | --- | --- | --- |
| **De Bruin et al, 2009, [25]**  *(Hodgkin lymphoma)* |  | 65/2201 | 2.2 (1.7-2.8) | 12 (7-18) |  |
| **Dorresteijn et al, 2001, [26]**  *(Head & Neck cancer)* |  | 14/367 |  | 3.8 | 5.6 (3.1-9.4) |
| **Hooning et al, 2006, [28]**  *(Breast cancer)* | Dutch female population | 164/4368 | 0.75 (0.64-0.88) | -15.2 per 10,000 patients per year |  |
| **Maduro et al, 2010, [33]**  *(Cervical cancer)* | Dutch reference population | 8/277 (2.9%) | 0.65 (0.28-1.29) | -25.12 per 10000 patients per year |  |
| **Moser et al, 2006, [35]**  *(non-Hodgkin lymphoma)* |  | 11/441 | 1.5 (1.1-2.7) | 15 per 10000 person years |  |
| **Nilsson et al, 2015, [12]**  *(Breast cancer)* |  | 1766/25171 |  |  | 1.12 (1.07-1.17) |
| **van-Hemelrijck et al, 2010, [44]**  *(Prostate cancer)* |  |  |  |  |  |
| *Endocrine treatment* | 2619/30642 |  | 1.26 (1.21-1.30) |  |  |
| *Curative treatment* | 1035/26432 |  | 0.98 (0.92-1.04) |  |  |
| *Surveillance* | 1923/19526 |  | 1.19 (1.14-1.25) |  |  |
| **Zoller et al,2012, [6]** |  |  |  |  |  |
| *Ischaemic stroke - All* | 396702 of Swedish population | 31524/820491 | 1.2 (1.2-1.2) |  |  |
| *Upper aerodigestive tract* |  | 776/820491 | 1.3 (1.2-1.4) |  |  |
| *Salivary gland* |  | 78/820491 | 1.3 (1.0-1.6) |  |  |
| *Oesophagus* |  | 81/820491 | 1.1 (0.9-1.4) |  |  |
| *Stomach* |  | 471/820491 | 1.2 (1.1-1.4) |  |  |
| *Small intestine* |  | 99/820491 | 1.1 (0.9-1.3) |  |  |
| *Colon* |  | 2786/820491 | 1.3 (1.2-1.3) |  |  |
| *Rectum* |  | 1430/820491 | 1.1 (1.1-1.2) |  |  |
| *Anus* |  | 66/820491 | 1.1 (0.8-1.4) |  |  |
| *Liver* |  | 158/820491 | 1.2 (1.0-1.4) |  |  |
| *Pancreas* |  | 139/820491 | 1.6 (1.4-1.9) |  |  |
| *Nose* |  | 59/820491 | 1.4 (1.1-1.8) |  |  |
| *Lung* |  | 809/820491 | 1.4 (1.3-1.5) |  |  |
| *Breast* |  | 4551/820491 | 1.1 (1.1-1.1) |  |  |
| *Cervix* |  | 217/820491 | 1.2 (1.1-1.4) |  |  |
| *Endometrium* |  | 1175/820491 | 1.1 (1.0-1.1) |  |  |
| *Ovary* |  | 392/820491 | 1.1 (1.0-1.2) |  |  |
| *Other female genital* |  | 148/820491 | 1.3 (1.1-1.5) |  |  |
| *Prostate* |  | 7495/820491 | 1.2 (1.2-1.2) |  |  |
| *Testis* |  | 32/820491 | 0.7 (0.5-1.0) |  |  |
| *Other male genital* |  | 64/820491 | 1.1 (0.9-1.4) |  |  |
| *Kidney* |  | 703/820491 | 1.1 (1.1-1.2) |  |  |
| *Urinary bladder* |  | 2269/820491 | 1.2 (1.2-1.3) |  |  |
| *Eye* |  | 68/820491 | 0.9 (0.7-1.1) |  |  |
| *Nervous system* |  | 642/820491 | 1.4 (1.3-1.6) |  |  |
| *Thyroid gland* |  | 171/820491 | 1.1 (1.0-1.3) |  |  |
| *Endocrine glands* |  | 831/820491 | 1.3 (1.2-1.4) |  |  |
| *Bone* |  | 20/820491 | 0.8 (0.5-1.3) |  |  |
| *Connective tissue* |  | 155/820491 | 1.1 (0.9-1.3) |  |  |
| *Non-Hodgkin lymphoma* |  | 1238/820491 | 1.1 (1.1-1.2) |  |  |
| *Hodgkin’s disease* |  | 66/820491 | 1.3 (1.0-1.6) |  |  |
| *Myeloma* |  | 276/820491 | 1.1 (1.0-1.2) |  |  |
| *Leukaemia* |  | 385/820491 | 1.5 (1.3-1.6) |  |  |
| *Haem stroke - All* | 77031 of Swedish population | 6926/820491 | 1.4 (1.3-1.4) |  |  |
| *Upper aerodigestive tract* |  | 148 | 1.3 (1.1-1.5) |  |  |
| *Salivary gland* |  | 17 | 1.5 (0.9-2.4) |  |  |
| *Oesophagus* |  | 22 | 1.6 (1.0-2.4) |  |  |
| *Stomach* |  | 116 | 1.6 (1.4-2.0) |  |  |
| *Small intestine* |  | 28 | 1.6 (1.0-2.2) |  |  |
| *Colon* |  | 567 | 1.4 (1.3-1.5) |  |  |
| *Rectum* |  | 289 | 1.2 (1.1-1.4) |  |  |
| *Anus* |  | 17 | 1.5 (0.9-2.4) |  |  |
| *Liver* |  | 39 | 1.6 (1.1-2.1) |  |  |
| *Pancreas* |  | 21 | 1.4 (0.8-2.1) |  |  |
| *Nose* |  | 17 | 2.1 (1.2-3.4) |  |  |
| *Lung* |  | 131 | 1.2 (1.0-1.4) |  |  |
| *Breast* |  | 721 | 1.0 (0.9-1.1) |  |  |
| *Cervix* |  | 33 | 1.0 (0.7-1.4) |  |  |
| *Endometrium* |  | 186 | 1.0 (0.9-1.2) |  |  |
| *Ovary* |  | 68 | 1.1 (0.8-1.4) |  |  |
| *Other female genital* |  | 21 | 1.1 (0.7-1.7) |  |  |
| *Prostate* |  | 1684 | 1.3 (1.2-1.4) |  |  |
| *Testis* |  | 16 | 1.4 (0.8-2.2) |  |  |
| *Other male genital* |  | 11 | 0.9 (0.5-1.7) |  |  |
| *Kidney* |  | 237 | 2.0 (1.8-2.3) |  |  |
| *Urinary bladder* |  | 435 | 1.2 (1.1-1.3) |  |  |
| *Eye* |  | 18 | 1.2 (0.7-2.0) |  |  |
| *Nervous system* |  | 368 | 4.2 (3.8-4.6) |  |  |
| *Thyroid gland* |  | 32 | 1.1 (0.8-1.6) |  |  |
| *Endocrine glands* |  | 201 | 1.7 (1.5-2.0) |  |  |
| *Bone* |  | 9 | 1.9 (0.9-3.7) |  |  |
| *Connective tissue* |  | 36 | 1.3 (0.9-1.8) |  |  |
| *Non-Hodgkin lymphoma* |  | 268 | 1.3 (1.2-1.5) |  |  |
| *Hodgkin’s disease* |  | 17 | 1.6 (0.9-2.5) |  |  |
| *Myeloma* |  | 93 | 2.0 (1.6-2.4) |  |  |
| *Leukaemia* |  | 223 | 4.4 (3.8-5.0) |  |  |

RR=Relative Risk; CI=Confidence Interval

Supplementary Table 7. Incidence of stroke in studies using odds ratio.

| **Study** | **Control group** | **Cancer group** | **OR (95% CI)** |
| --- | --- | --- | --- |
| **van Herk-Sukel et al,2011, [45]**  *(Breast cancer)* | 85/114730 | 15/11473 | 1.8 (1.0-3.1) |
| **van Herk-Sukel et al, 2013, [46]**  *(Lung cancer)* | 60/37170 | 11/3717 | 1.8 (0.97-3.5) |

OR=Odds Ratio; CI=Confidence Interval
